# BNIP3 and NIX Mediate Mieap-Induced Accumulation of Lysosomal Proteins within Mitochondria

**DOI:** 10.1371/journal.pone.0030767

**Published:** 2012-01-26

**Authors:** Yasuyuki Nakamura, Noriaki Kitamura, Daisuke Shinogi, Masaki Yoshida, Olga Goda, Ryuya Murai, Hiroki Kamino, Hirofumi Arakawa

**Affiliations:** Division of Cancer Biology, National Cancer Center Research Institute, Tokyo, Japan; Roswell Park Cancer Institute, United States of America

## Abstract

Mieap, a p53-inducible protein, controls mitochondrial quality by repairing unhealthy mitochondria. During repair, Mieap induces the accumulation of intramitochondrial lysosomal proteins (designated MALM for Mieap-induced accumulation of lysosome-like organelles within mitochondria) by interacting with NIX, leading to the elimination of oxidized mitochondrial proteins. Here, we report that an additional mitochondrial outer membrane protein, BNIP3, is also involved in MALM. BNIP3 interacts with Mieap in a reactive oxygen species (ROS)-dependent manner via the BH3 domain of BNIP3 and the coiled-coil domains of Mieap. The knockdown of endogenous BNIP3 expression severely inhibited MALM. Although the overexpression of either BNIP3 or NIX did not cause a remarkable change in the mitochondrial membrane potential (MMP), the co-expression of all three exogenous proteins, Mieap, BNIP3 and NIX, caused a dramatic reduction in MMP, implying that the physical interaction of Mieap, BNIP3 and NIX at the mitochondrial outer membrane may regulate the opening of a pore in the mitochondrial double membrane. This effect was not related to cell death. These results suggest that two mitochondrial outer membrane proteins, BNIP3 and NIX, mediate MALM in order to maintain mitochondrial integrity. The physical interaction of Mieap, BNIP3 and NIX at the mitochondrial outer membrane may play a critical role in the translocation of lysosomal proteins from the cytoplasm to the mitochondrial matrix.

## Introduction

Mieap is a p53-inducible protein for which transcription is directly regulated by the tumor suppressor p53 [1]. We have recently reported that Mieap controls mitochondrial quality via two distinct mechanisms [1], [2]. One of the mechanisms has been designated MALM for Mieap-induced accumulation of lysosome-like organelles within mitochondria [1]. In this mechanism, Mieap induces the accumulation of intramitochondrial lysosomal proteins to eliminate oxidized mitochondrial proteins in response to mitochondrial damage. This leads to a decrease in reactive oxygen species generation and an increase in mitochondrial ATP synthesis activity. Therefore, this function likely mediates the repair of unhealthy mitochondria. Alternatively, the other mechanism has been designated MIV for Mieap-induced vacuole [2]. When MALM is inhibited, Mieap induces a vacuole-like structure known as the MIV. The MIV engulfs the damaged mitochondria and accumulates lysosomes, leading to the degradation of unhealthy mitochondria. The function of the MIV is likely to be a type of mitochondrial autophagy. Therefore, Mieap controls mitochondrial quality by repairing or eliminating unhealthy mitochondria via MALM or MIV generation, respectively [1], [2]. The inactivation of p53 or Mieap severely impairs both MALM and MIV, leading to the accumulation of unhealthy mitochondria [1], [2]. Although Mieap-mediated mitochondrial quality control appears to be critical for a variety of diseases and biological responses, including aging, cancer, and degenerative diseases, a large part of the mechanism still remains to be elucidated.

BNIP3 is a BH3-only protein that belongs to the Bcl-2 family [3]. Initially, BNIP3 was identified as a Bcl-2-interacting protein using yeast two-hybrid screening, and it was suggested to induce apoptosis by inhibiting anti-apoptotic proteins, including Bcl-2 and Bcl-xL [4]. However, subsequent studies have reported that BNIP3-induced cell death is not inhibited by caspase inhibitors and that the release of cytochrome c is not required for BNIP3-induced cell death [5], [6]. Furthermore, in contrast to other proapoptotic BH3-only proteins, including Puma and Noxa, the BH3 domain of BNIP3 is not required for the induction of cell death [6], [7]. These facts suggest that BNIP3 may have a unique function that is distinct from the other BH3-only proteins. Interestingly, only NIX (also designated BNIP3L), which shares 55% sequence homology with BNIP3, has been reported to have a similar phenotype during cell death induction [3]. The cell death induced by BNIP3 and NIX involves the opening of mitochondrial permeability transition pores, leading to mitochondrial depolarization and necrosis-like cell death [3], [5]. This unique role of BNIP3 and NIX in cell death has mainly been thought to be important for cardiovascular diseases [8]. The physiological functions of BNIP3 and NIX appear to be complicated and largely remain to be elucidated. In particular, the role of the BH3 domains of BNIP3 and NIX is unclear.

Here, we report that BNIP3 and NIX play pivotal roles in the Mieap-regulated mitochondrial quality control. BNIP3 and NIX interact with Mieap at the mitochondrial outer membrane, mediating the translocation of lysosomal proteins into the mitochondria. The BH3 domains of BNIP3 and NIX are critical for their interactions with Mieap. The interaction of Mieap, BNIP3, and NIX at the mitochondrial outer membrane may open a pore that spans from the mitochondrial outer membrane to the inner membrane in order to induce MALM.

## Results

### BNIP3 and NIX interact with Mieap

Previously, we reported that NIX interacts with Mieap at the mitochondrial outer membrane (MOM) and mediates the induction of MALM [2]. Therefore, we speculated that BNIP3, a homolog of NIX, may also interact with Mieap at the MOM. To evaluate this hypothesis, we examined the interaction of endogenous BNIP3 and Mieap proteins using fractionated mitochondria derived from γ-irradiated A549 control (cont) and Mieap-knockdown (Mieap-KD) cells. The control and Mieap-KD A549 cells were irradiated using γ-rays, and the mitochondria were isolated 3 days after ionizing radiation (IR). We confirmed that MALM was induced in the A549 control cells, but not in the Mieap-KD cells, as previously reported (data not shown). We also confirmed that the expression levels of NIX and BNIP3 were increased in the A549 control cells, but not in the NIX-KD and BNIP3-KD cells on day 3 after IR (Figure S1).

**Figure 1:**
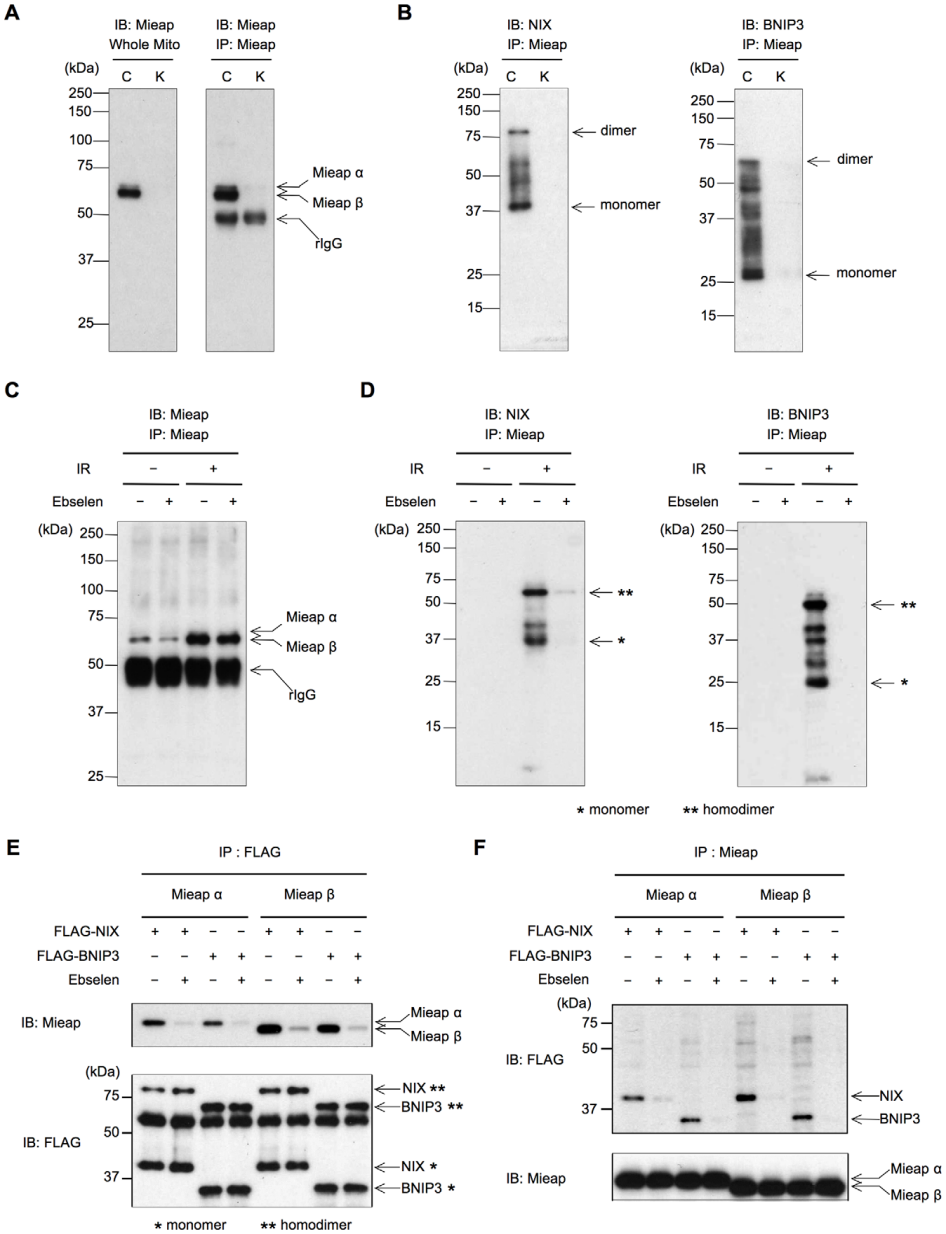
BNIP3 and NIX interact with Mieap. (A and B) The interaction between endogenous Mieap and NIX/BNIP3. The control A549 cells were γ-irradiated, and 2 days later, the cell lysates were purified from the mitochondrial fraction. (A) The endogenous Mieap protein was precipitated from the lysates using an anti-Mieap antibody. The precipitated proteins were subjected to western blot analysis using an anti-Mieap antibody. (B) Endogenous BNIP3 and NIX interact with endogenous Mieap. The same blot as in (A) was subjected to immunoblotting using anti-NIX and anti-BNIP3 antibodies. (C and D) The interaction between endogenous Mieap and NIX/BNIP3 is ROS-dependent. The same experiment as (A and B) was performed with the A549-control cells in the presence or absence of Ebselen. IR: ionizing radiation. (E and F) Exogenous BNIP3 and NIX interact with exogenous Mieap. HCT116 cells were transfected with plasmids expressing FLAG-BNIP3 or FLAG-NIX and infected with Ad-Mieap α or Ad-Mieap β in the presence or absence of Ebselen. The cells were subjected to IP analysis 2 h after the infection and transfection. The NIX and BNIP3 proteins were precipitated using an anti-FLAG antibody (E), and the Mieap α and Mieap β proteins were precipitated using an anti-Mieap antibody (F). The precipitates were subjected to western blot analysis using anti-Mieap and anti-FLAG antibodies. IP: immunoprecipitation, IB: immunoblot, FLAG: anti-FLAG antibody, Mieap: anti-Mieap antibody.

As shown in Figure 1A, endogenous Mieap α and β proteins were precipitated using an anti-Mieap antibody. Interestingly, compared with Mieap α, the Mieap β protein was preferentially present in the γ-irradiated mitochondria and predominantly precipitated from the fractionated mitochondria, suggesting a specific role for Mieap β in MALM. Immunoblotting of the same precipitates using anti-NIX or anti-BNIP3 antibodies clearly indicated that endogenous BNIP3 and NIX proteins were included in the Mieap protein complex, suggesting that they interact with Mieap at the mitochondria (Figure 1B).

We previously reported that ROS scavengers inhibits MALM, suggesting a pivotal role of ROS in the MALM mechanism [1], [2]. Therefore, we examined the role of ROS in the interaction between endogenous Mieap and NIX/BNIP3 proteins using Ebselen. As shown in Figure 1C, the Mieap protein was significantly increased in the mitochondria of the A549 control cells after IR regardless of the presence or absence of Ebselen. Consistent with the results of Figure 1A and 1B, endogenous NIX and BNIP3 proteins were co-precipitated with Mieap after IR. However, the Ebselen treatment clearly inhibited the interaction between endogenous Mieap and NIX/BNIP3 proteins in the irradiated A549 control cells (Figure 1D). These results suggest that mitochondrial ROS play a critical role in the interaction between endogenous Mieap and NIX/BNIP3 proteins during the process of MALM.

To further confirm and characterize the interaction between Mieap and NIX/BNIP3, exogenous Mieap α, Mieap β, FLAG-NIX, and FLAG-BNIP3 proteins were expressed through infection using Ad-Mieap α or Ad-Mieap β and transfection with plasmids expressing FLAG-NIX or FLAG-BNIP3. The interactions between Mieap and BNIP3 or NIX were examined using immunoprecipitation (IP) experiments with anti-Mieap or anti-FLAG antibodies. As shown in Figure 1E, when either FLAG-BNIP3 or FLAG-NIX was immunoprecipitated using the anti-FLAG antibody, Mieap α and Mieap β were detected in the immunoprecipitates. Conversely, when either Mieap α or Mieap β was immunoprecipitated using the anti-Mieap antibody, both FLAG-BNIP3 and FLAG-NIX were detected in the precipitated protein complex using immunoblotting with the anti-FLAG antibody (Figure 1F). However, the interactions were dramatically inhibited by treatment with Ebselen, implying that ROS are critical for the interactions. These results suggest that BNIP3 and NIX interact with Mieap in a ROS dependent manner.

Consistent with the preferential localization of Mieap β at the mitochondria (Figure 1A and 1C), the Mieap β signal in the NIX and BNIP3 immunoprecipitates was much stronger than the Mieap α signal. Moreover, the NIX and BNIP3 signals appeared to be stronger in the Mieap β immunoprecipitates compared with the Mieap α immunoprecipitates (Figure 1F).

### The coiled-coil domains of Mieap are critical for the interactions with BNIP3 and NIX

**Figure 2:**
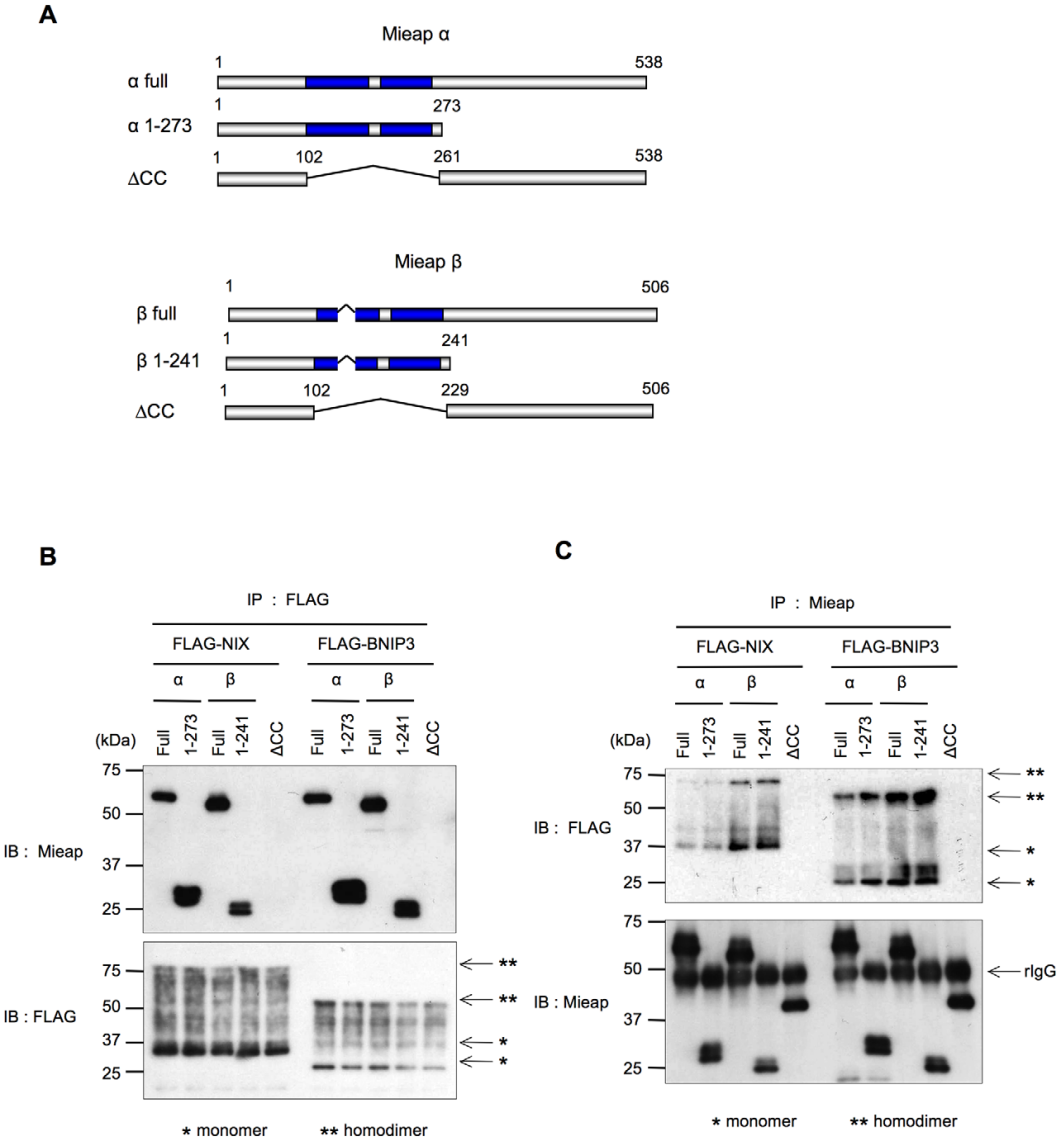
The coiled-coil domains of Mieap are critical for its interaction with BNIP3 and NIX. (A) The Mieap α and Mieap β mutant proteins. The adenoviral vectors expressing deletion mutants lacking the C-terminal region (α1–273 and β1–241) and the coiled-coil regions (ΔCC) of Mieap α and Mieap β proteins were prepared as indicated. (B–C) The mutants lacking coiled-coil regions of Mieap α and Mieap β proteins fail to bind NIX and BNIP3. HCT116 cells were transfected with plasmids expressing FLAG-NIX and FLAG-BNIP3 and infected with adenoviral vectors expressing the various Mieap α and Mieap β mutants. Cell lysates were isolated 2 days after transfection and infection and subjected to IP experiments. FLAG-NIX and FLAG-BNIP3 or the Mieap α and Mieap β mutants were precipitated using anti-FLAG (B) or anti-Mieap (C) antibodies, respectively. The precipitates were subjected to western blot analysis using anti-Mieap or anti-FLAG antibodies.

To determine which region of Mieap was critical for the interaction with BNIP3 and NIX, we prepared adenoviral vectors expressing various Mieap α and Mieap β mutants (Figure 2A) and performed IP experiments using these vectors.

As shown in Figure 2B, when FLAG-NIX and FLAG-BNIP3 were immunoprecipitated using the anti-FLAG antibody, the ΔCC (coiled-coil) mutants of Mieap α and Mieap β failed to bind to BNIP3 and NIX, whereas the Δ274–538 Mieap α (α1–273) and Δ242–506 Mieap β (β1–241) mutants were able to interact with BNIP3 and NIX. These results were validated when the Mieap mutants were immunoprecipitated using the anti-Mieap antibody (Figure 2C). These results suggest that the coiled-coil domains of Mieap α and Mieap β are critical for the interactions with BNIP3 and NIX.

Furthermore, we observed that the NIX and BNIP3 signals were much stronger in the Mieap β immunoprecipitates than in the Mieap α immunoprecipitates (Figure 2C). These results also suggest that compared with Mieap α, Mieap β may preferentially interact with BNIP3 and NIX.

### The BH3 domains of BNIP3 and NIX are critical for the interaction with Mieap

**Figure 3:**
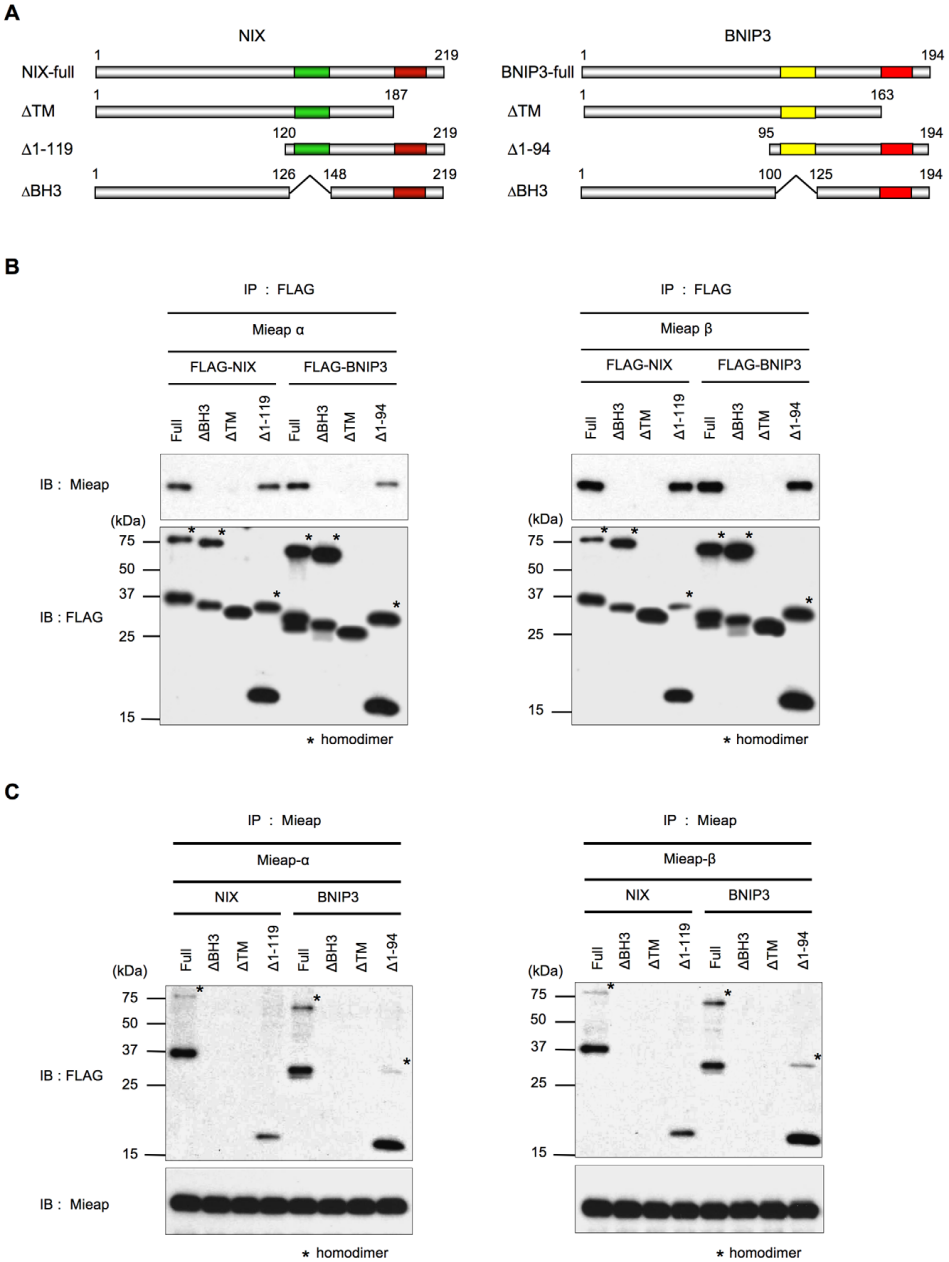
The BH3 domains of BNIP3 and NIX are critical for the interaction with Mieap. (A) The mutant NIX and BNIP3 proteins. The plasmids expressing deletion mutants lacking the transmembrane domain (ΔTM), the N-terminal region (Δ1–119 for NIX and Δ1–94 for BNIP3), and the BH3 region (ΔBH3) of NIX and BNIP3 proteins were prepared as indicated. (B and C) The NIX and BNIP3 mutants lacking the BH3 or TM domains fail to bind Mieap. HCT116 cells were transfected with plasmids expressing the various FLAG-NIX and FLAG-BNIP3 mutants, and the cells were infected with adenoviral vectors expressing Mieap α and Mieap β. Cell lysates were isolated 2 days after transfection and infection and subjected to IP experiments. The various FLAG-NIX and FLAG-BNIP3 mutants or Mieap α and Mieap β were precipitated using anti-FLAG (B) or anti-Mieap (C) antibodies, respectively. The precipitates were subjected to western blot analysis using anti-Mieap or anti-FLAG antibodies.

To determine which regions of BNIP3 and NIX were responsible for the interaction with Mieap, we prepared plasmids expressing various BNIP3 and NIX mutants (Figure 3A) and performed IP experiments using these constructs.

As shown in Figure 3B, when the BNIP3 mutants were precipitated using the anti-FLAG antibody, the ΔBH3 and ΔTM mutants were unable to bind to either Mieap α or Mieap β, whereas the full-length protein and the Δ1–119 mutant could bind to both Mieap α and Mieap β. Similar results were obtained using the NIX mutants (Figure 3B). These results were validated using immunoprecipitation of the Mieap α and Mieap β proteins using the anti-Mieap antibody (Figure 3C).

Because our previous study indicated that the TM domain of NIX was critical for its mitochondrial localization (2), we further examined the subcellular localization of the BNIP3 mutants using immunofluorescence (IF) experiments. As indicated in Figure S2, the ΔBH3 mutant was consistently detected at the mitochondria, whereas the ΔTM mutant was unable to localize at the mitochondria because of the lack of the C-terminal TM domain. Therefore, we conclude that the BH3 domain of BNIP3 plays a critical role in the interaction with Mieap at the MOM.

### Mieap interacts with BNIP3 and NIX at the mitochondrial outer membrane

**Figure 4:**
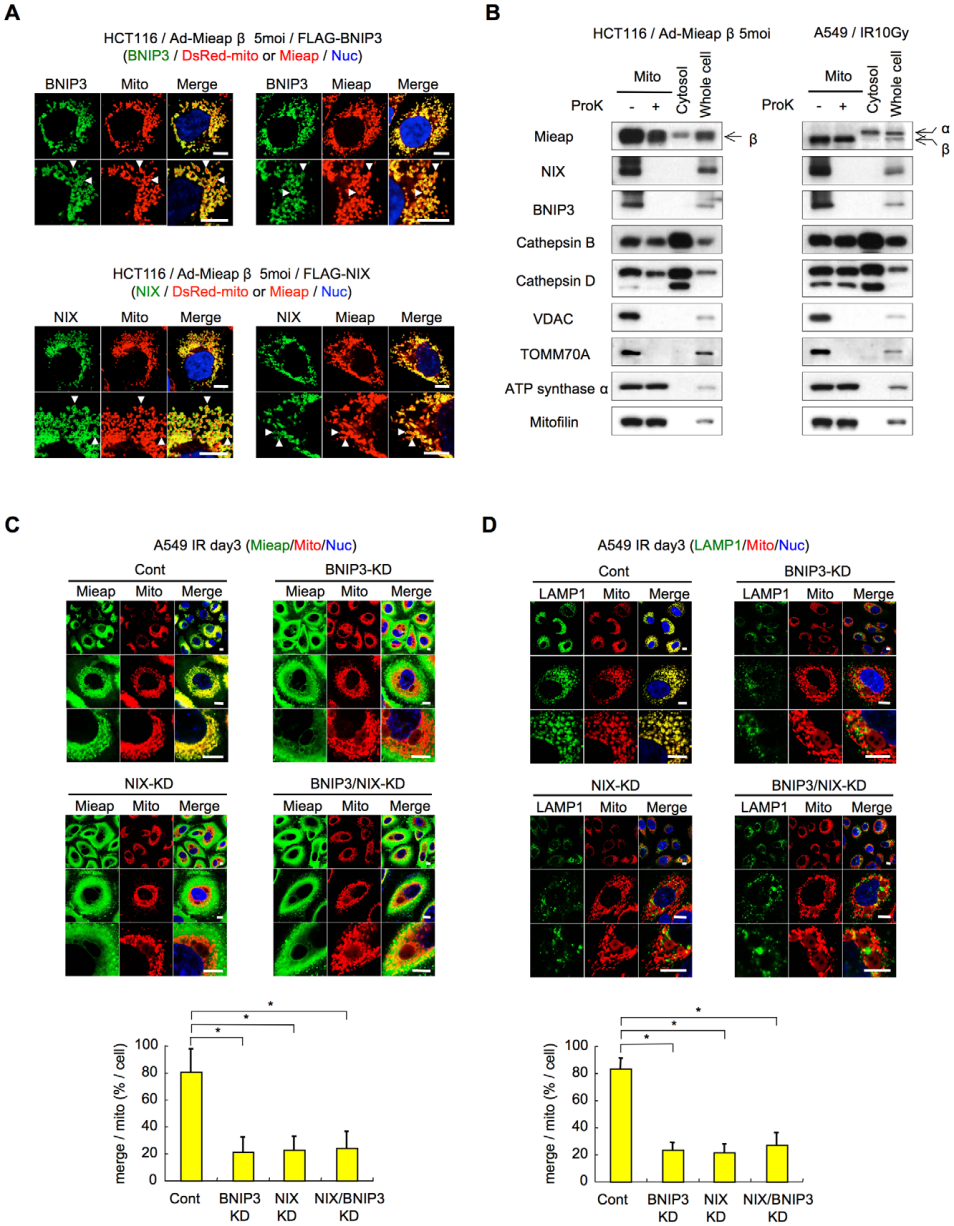
BNIP3 and NIX interact with Mieap at the mitochondrial outer membrane to mediate MALM. (A) Immunofluorescence (IF) experiments. HCT116 cells were infected with Ad-Mieap β and Ad-DsRed-mito at an MOI of 5 and transfected with plasmids expressing FLAG-BNIP3 or FLAG-NIX. After 2 days, the cells were subjected to IF analysis using the anti-Mieap antibody (red) and the anti-FLAG antibody (green). The mitochondria were detected using DsRed-mito (red). The arrowheads indicate the representative data for BNIP3, NIX, DsRedmito, and Mieap. Scale bar = 10 µm. (B) Proteinase K protection assay. HCT116 cells were infected with Ad-Mieap β at an MOI of 5, and A549 and LS174T cells were γ-irradiated. The mitochondria were fractionated from the Ad-Mieap β infected HCT116 cells or the irradiated A549 cells 2 days after infection or radiation. The proteinase K protection assay was performed using the mitochondrial fractions. (C) BNIP3 deficiency inhibits the mitochondrial localization and accumulation of Mieap. A549-cont, BNIP3-KD, NIX-KD, and BNIP3/NIX-KD cells were γ-irradiated, and 3 days after IR, the cells were subjected to IF analysis. The Mieap protein was stained using the anti-Mieap antibody (Mieap: green). Mitochondria were identified using the DsRed-mito protein signal (Mito: red). The yellow areas indicate the mitochondrial localization and accumulation of Mieap. Quantitative analysis of the yellow and red areas was performed in 300–400 cells. The average values for the ratio of yellow to red signals (merged/mitochondrial; yellow bar graph) are presented; the error bars indicate 1 SD. p<0.01 (*) was considered statistically significant. Scale bar = 10 µm. (D) BNIP3 deficiency inhibits MALM. Using the protocol described in (A), A549-cont, BNIP3-KD, NIX-KD, and BNIP3/NIX-KD cells were also analyzed for MALM. Lysosomes were stained using the anti-LAMP1 antibody (LAMP1: green), and mitochondria were identified using the DsRed-mito protein signal (Mito: red). The yellow areas indicate the accumulation of lysosomes within mitochondria. Quantitative analysis of the yellow and red areas was performed according to the procedure described in panel (C). The average values of the ratio of yellow to red areas (merged/mitochondrial; yellow bar graph) are presented; the error bars indicate 1 SD. p<0.01 (*) was considered statistically significant. Scale bar = 10 µm.

To determine where Mieap interacted with BNIP3 and NIX in the cell, we performed IF experiments. As indicated in Figure 4A, the BNIP3 signal was localized at the marginal region of the mitochondria, suggesting that BNIP3 localizes to the MOM. Consistent with this result, double-staining for Mieap and BNIP3 indicated that Mieap colocalized with BNIP3 at the MOM (yellow signal), whereas Mieap was also detected within the mitochondria (red signal). We obtained a similar result with NIX (Figure 4A).

To further confirm the IF results, we performed proteinase K protection assays. The localization of Mieap, BNIP3, and NIX was examined in Ad-Mieap β-infected HCT116 cells (exogenous proteins) and γ-irradiated A549 and LS174T cells (endogenous proteins). MALM was induced, and the mitochondria were isolated from the individual cell lines and subjected to the assay. As shown in Figure 4B, BNIP3 and NIX were degraded after proteinase K treatment in parallel with the two mitochondrial outer membrane proteins VDAC and TOMM70A. However, as previously reported, Mieap and the two lysosomal proteins cathepsin B and cathepsin D were protected from proteinase K digestion, similar to the two intramitochondrial proteins ATP synthase α and mitofilin. These results suggest that Mieap interacts with BNIP3 and NIX at the MOM.

### BNIP3 and NIX are essential for MALM

We have previously reported the interaction of NIX and Mieap and its critical role in MALM [2]. To evaluate the significance of the interaction between BNIP3 and Mieap, we examined the effect of BNIP3 knockdown on MALM. The BNIP3 expression was significantly inhibited in the BNIP3-KD and BNIP3/NIX-KD A549 cells (Figure S3). As shown in Figure 4C, inhibiting BNIP3 expression in A549-BNIP3-KD cells dramatically impaired the mitochondrial localization of Mieap. Moreover, the knockdown of BNIP3 expression almost completely inhibited MALM (Figure 4D). Similar results were obtained in NIX knockdown cells. Interestingly, the double knockdown of BNIP3 and NIX in A549 cells did not enhance the effect of the single knockdown of BNIP3 or NIX in the same cells, implying that BNIP3 and NIX are equally required for the induction of MALM in the same pathway. These results suggest that both BNIP3 and NIX are essential factors for MALM.

### The interaction of Mieap, BNIP3, and NIX may regulate the opening of a pore in the mitochondrial double membrane to induce MALM but not cell death

Because BNIP3 and NIX have been reported to play an important role in cell death via the regulation of the mitochondrial permeability transition pore (MPTP) [3], we previously speculated that BNIP3 and/or NIX may regulate or form a large hole (here designated as the Mieap-Regulated Pore (MRP)) that spans from the mitochondrial outer membrane to the inner membrane, likely leading to the translocation of lysosomes into the mitochondrial matrix in a process regulated by Mieap and MALM induction [1], [2].

Because the mitochondrial membrane potential (MMP) is reduced by the influx of H^+^ from the cytoplasm to the mitochondrial matrix because of the opening of a pore in the mitochondrial double membrane, we assumed that the effect on MMP reflected the status of the opening of MRP in the mitochondrial double membrane. To evaluate the role of NIX and BNIP3 in the opening of the MRP, we examined the effect of the expression of exogenous BNIP3, NIX, and Mieap on MMP. To examine the role of these interactions in MALM, HCT116 and A549 cells were infected with Ad-Mieap α or Ad-Mieap β at an MOI of 5, which is a relatively low dose of adenovirus, to induce MALM. BNIP3 and NIX were also expressed through infection with Ad-BNIP3 and Ad-NIX at an MOI of 5.

**Figure 5:**
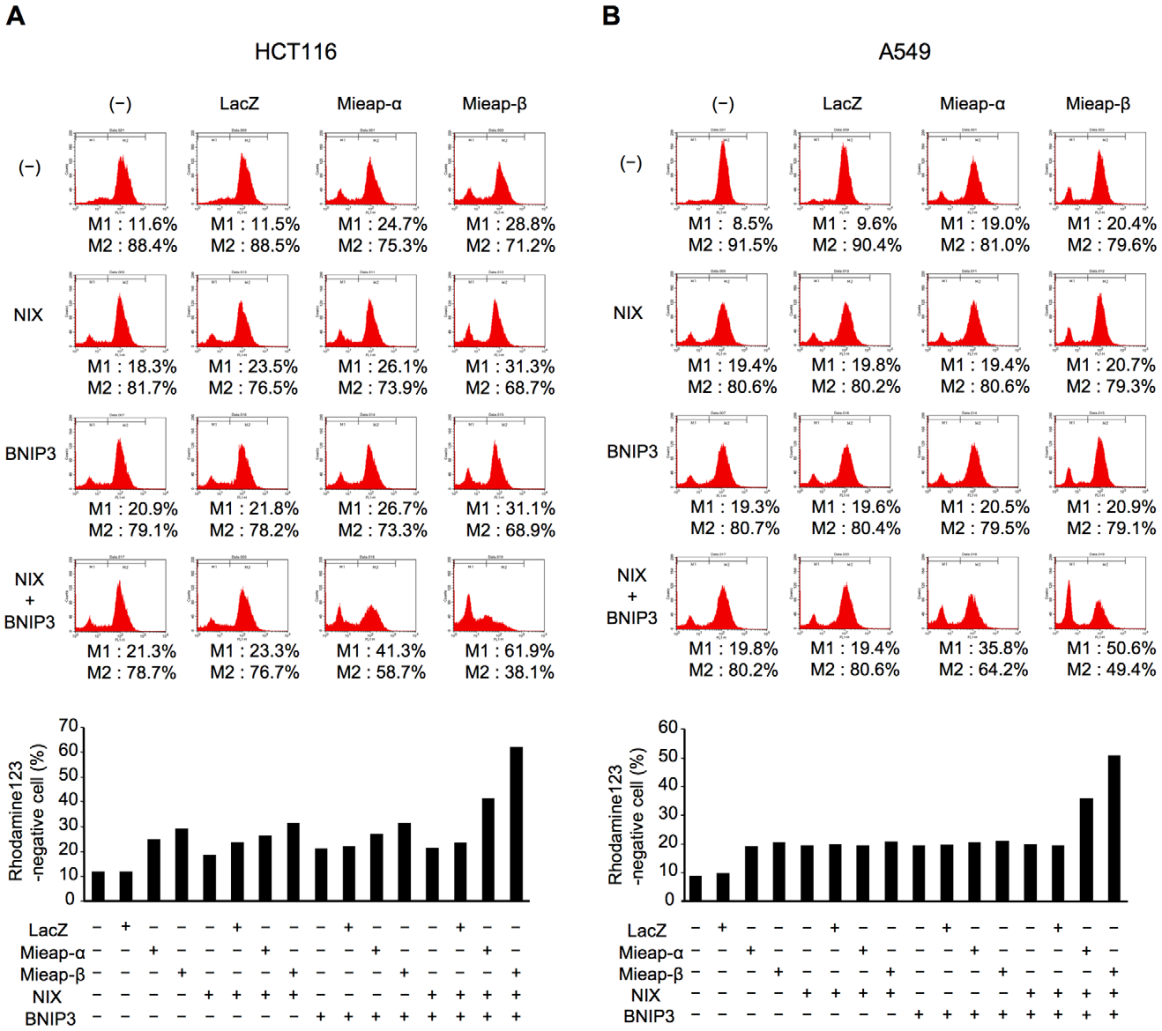
The triple co-expression of Mieap, BNIP3, and NIX induces a remarkable reduction of MMP. HCT116 (A) and A549 (B) cells were infected with the indicated combinations of Ad-LacZ, Ad-NIX, Ad-BNIP3, Ad-Mieap α, and Ad-Mieap β, and 2 days after the infection, FACS analysis was performed to examine the MMP status. The experiment was repeated three times, and the representative result was shown. M1: rhodamine negative cells, M2: rhodamine positive cells. The experiment was independently carried out three times, and the representative result was shown.

As shown in Figure 5, the expression of either Mieap α or Mieap β alone through infection with Ad-Mieap α or Ad-Mieap β induced a slight decrease in MMP (M1: cont 11.6% and LacZ 11.5% vs Mieap α 24.7% and Mieap β 28.8%). The infection with Ad-BNIP3, Ad-NIX or both Ad-BNIP3 and Ad-NIX also resulted in a small decrease in MMP (M1: cont 11.6% and LacZ 11.5% vs BNIP3 20.9% and NIX 18.3%). Additionally, the co-infection with Ad-BNIP3 and Ad-Mieap α or Ad-Mieap β or the co-infection with Ad-NIX and Ad-Mieap α or Ad-Mieap β did not enhance the effect of the expression of BNIP3, NIX or Mieap alone. However, the triple co-expression of BNIP3, NIX, and Mieap α or BNIP3, NIX, and Mieap β led to a dramatic reduction in MMP (Figure 5). These results suggest that the interaction of the three proteins, Mieap, NIX, and BNIP3, may induce a pore in the mitochondrial double membrane. We further observed that Mieap β enhanced the effect of NIX and BNIP3 much more dramatically than Mieap α, supporting the idea that Mieap β may have a specific role in the regulation of MALM.

BNIP3 and NIX have been reported to induce cell death by reducing MMP via the regulation of MPTP [3]. However, we did not observe dead cells in the above experiment using Ad-BNIP3 and Ad-NIX at an MOI of 5 (Figure 5). To exclude the possibility that NIX and BNIP3 were not sufficiently expressed by the infection with the low dose of adenovirus vector, we increased the NIX and BNIP3 expression levels by infection with Ad-BNIP3 and Ad-NIX at the MOIs of 30 and 100. Although NIX and BNIP3 were actually overexpressed in HCT116 cells by infection with the high dose of Ad-BNIP3 and Ad-NIX (Figure S4), we observed neither of the MMP reduction (Figure S5A), the increased dead cells (Figures S5B and S6), nor the caspase-3 activation (Figure S5C) at both early (48 h after infection) and late phases (98 h after infection). In contrast, the infection with Ad-p53 at a MOI of 5 as a positive control dramatically induced the MMP reduction (Figure S5A), the increased dead cells (Figures S5B and S6), and the caspase-3 activation (Figure S5C).

**Figure 6:**
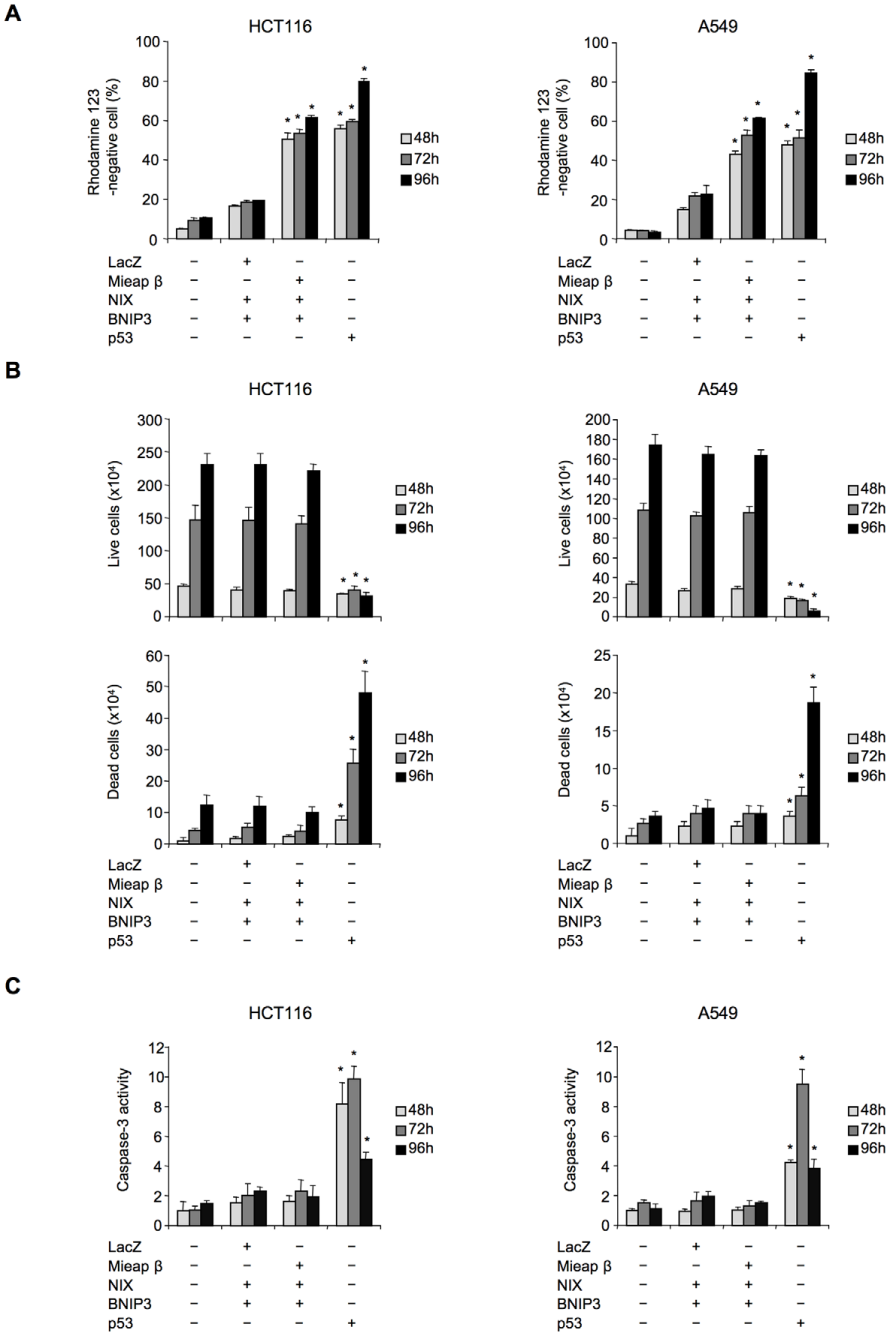
The MMP reduction induced by the triple co-expression of Mieap, BNIP3, and NIX is not related to cell death. HCT116 and A549 cells were co-infected with the adenoviral vectors Ad-LacZ, Ad-NIX, and Ad-BNIP3 or Ad-Mieap β, Ad-NIX, and Ad-BNIP3 at a MOI of 5. At 48 h, 72 h, 98 h after infection, the reduction of MMP was determined through the percentage of rhodamine-negative cells (A), and cell death was evaluated by trypan blue exclusion assay (B), and by measuring the caspase-3 activity (C). Ad-p53 at a MOI of 5 was used as a positive control for cell death. The average values of three independent experiments are presented; the error bars indicate 1 SD. p<0.01 (*) was considered statistically significant between the triple infection of Ad-LacZ, Ad-NIX, and Ad-BNIP3 and that of Ad-Mieap, Ad-NIX, and Ad-BNIP3 (A), or the infection of Ad-p53 (A, B, and C).

Because the co-expression of Mieap, NIX, and BNIP3 by co-infection with Ad-Mieap, Ad-BNIP3, and Ad-NIX at a MOI of 5 resulted in the dramatic reduction of MMP, we reasoned that cell death must be induced under this condition. Surprisingly, although MMP was dramatically reduced in response to the co-expression of the three proteins (Figure 6A), we found neither of dead cells (Figure 6B and Figure S7), caspase-3 activation (Figure 6C), nor the cytochrome c release from mitochondria (Figure S8) in any of HCT116 and A549 cells from early phase (48 h after infection) to late phase (96 h after infection). In contrast, the infection with Ad-p53 at a MOI of 5 dramatically induced not only the reduction in MMP (Figure 6A) but also the increased dead cells (Figure 6B and Figure S7), the activation of caspase-3 (Figure 6C), and the release of cytochrome c from the mitochondria (Figure S8). These results suggest that the reduction in MMP by these three proteins is not related to cell death at all; however, the interaction between these three proteins at the MOM may open a pore in the mitochondrial double membrane for MALM.

**Figure 7:**
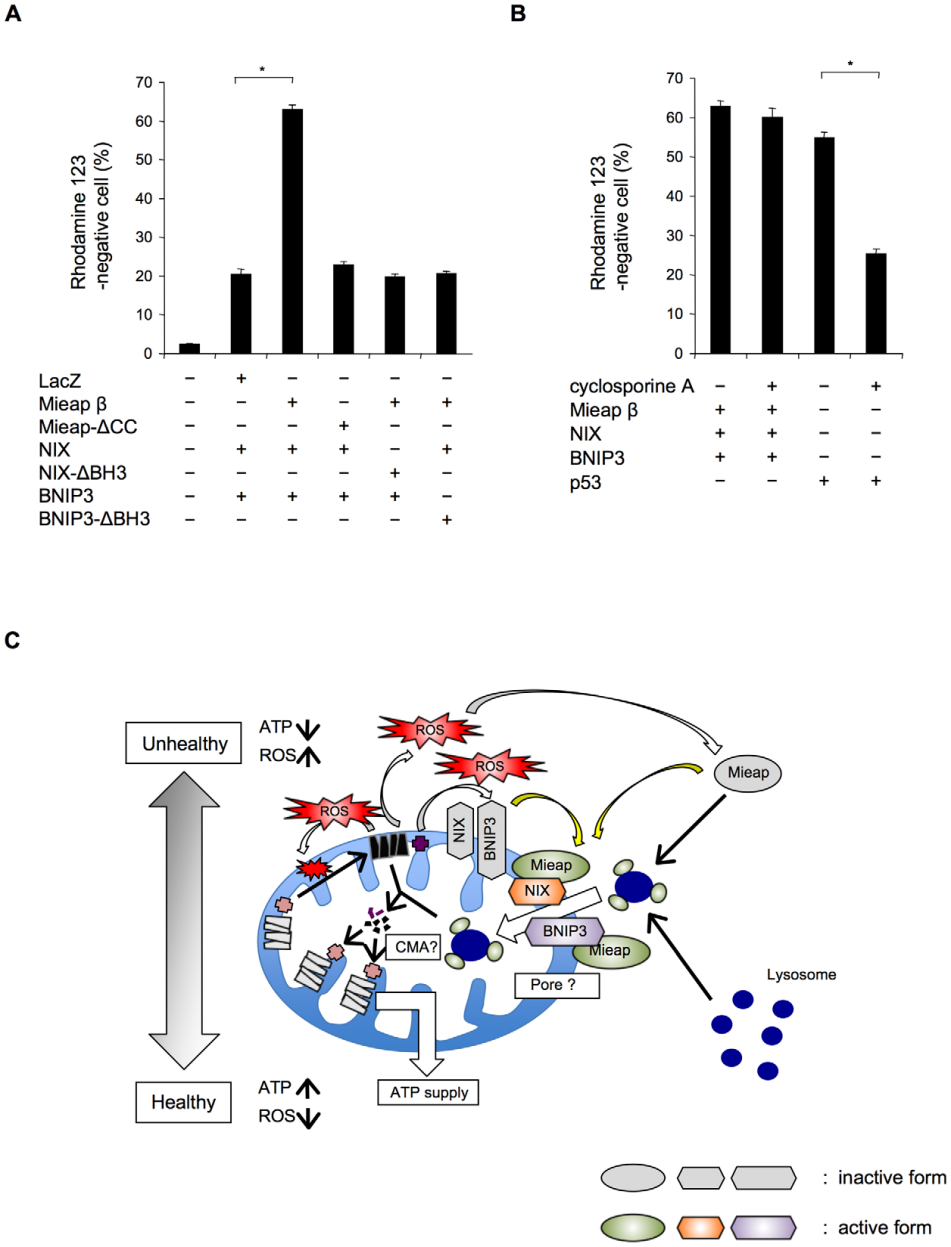
The physical interaction of Mieap, NIX, and BNIP3 at mitochondrial outer membrane regulates the opening a pore in the mitochondrial double membrane to induce MALM. (A) The physical interaction between the three proteins is critical for the opening a pore. The status of MMP was examined at 48 h after the triple infection with either of Ad-Mieap *β* ΔCC, Ad-NIX, and Ad-BNIP3, or Ad-Mieap *β*, Ad-NIX ΔBH3, and Ad-BNIP3, or Ad-Mieap *β*, Ad-NIX, and Ad-BNIP3 ΔBH3. p<0.01 (*) was considered statistically significant. (B) The pore induced by the three proteins interaction is not MPTP. The status of MMP was examined in the presence or absence of cyclosporine A at 48 h after the triple infection of Ad-Mieap *β*, Ad-NIX, and Ad-BNIP3, or the infection of Ad-p53. p<0.01 (*) was considered statistically significant. (C) Hypothetical model for MALM. The unhealthy mitochondria produce high levels of ROS, leading to activation of Mieap, NIX, and BNIP3. The activated forms of Mieap, NIX, and BNIP3 interact with one another at mitochondrial outer membrane, resulting in the opening a pore in the mitochondrial double membrane. The pore mediates the translocation of lysosomal proteins from the cytoplasm to the mitochondrial matrix.

As shown in Figures 2 and 3, the CC domains of Mieap and the BH3 domains of NIX and BNIP3 are critical for the interaction between Mieap and NIX/BNIP3. Using the Mieap, NIX, and BNIP3 mutants lacking the CC domains or the BH3 domain, we further examined the role of the three proteins interaction in the pore opening. As shown in Figure 7A, co-expression of three proteins including either of the Mieap-ΔCC, NIX-ΔBH3, or BNIP3-ΔBH3 mutant failed to induce the reduction in MMP, implying that the interaction between three proteins at the MOM are critical for opening the pore in the mitochondrial double membrane.

We finally characterized the nature of the pore induced by the three proteins interaction. Because NIX and BNIP3 were reported to regulate MPTP in order to reduce MMP and cyclosporine A is known to inhibit MPTP, we examined the effect of cyclosporine A on the pore induced by three proteins. Although the MMP reduction induced by p53 was severely inhibited by cyclosporine A, the reduction of MMP by three proteins was not affected by cyclosporine A (Figure 7B). This result suggests that the pore induced by the interaction between Mieap, NIX and BNIP3 is not MPTP.

## Discussion

Since the discovery of BNIP3 and NIX, a number of studies have been performed to clarify the role of BNIP3 and NIX in cell death, and several important and specific findings have been reported. According to the previous reports, the cell death induced by BNIP3 and NIX is characterized by atypical phenotypes. First, cell death is likely to be independent of cytochrome c release and caspase activation, implying that necrosis-like cell death, not apoptosis, is occurring [5]. Second, in contrast to other BH3-only proteins, the TM domain, not the BH3 domain, is critical for the induction of cell death [9]–[13]. Third, BNIP3 and NIX reduce MMP by regulating the MPTP [5], [10], [11]. Fourth, the ability of BNIP3 and NIX to induce cell death is much weaker than the other BH3-only proteins [14]. These facts suggest that BNIP3 and NIX may have additional functions that are unrelated to cell death.

Consistent with their reported weak ability to induce cell death, in the present study we did not observe any induction of apoptosis, non-apoptotic cell death or significant changes in MMP in response to BNIP3 or NIX overexpression in any of the two cell lines examined. However, we did observe a remarkable decrease in MMP only when the three exogenous proteins Mieap, BNIP3 and NIX were co-expressed in the same cells, implying that three proteins may cooperate with one another in order to open a pore in the mitochondrial double membrane. Surprisingly, even in this case, we never observed cell death, despite the remarkable reduction of MMP. This result suggests that the opening the pore by three proteins is not related to the induction of cell death but reflects another function.

We have previously speculated that BNIP3 and/or NIX may regulate or form a large hole that spans from the mitochondrial outer membrane to the inner membrane, leading to the translocation of lysosomal proteins into the mitochondrial matrix in a process regulated by Mieap through the induction of MALM [1], [2]. On the basis of the findings of this study, we speculate that the cooperation and interaction of three proteins Mieap, BNIP3, and NIX at the MOM may open a pore in the mitochondrial double membrane to induce MALM, which is not related to cell death induction (Figure 7C).

In fact, using the deletion mutants including Mieap-ΔCC, NIX-ΔBH3, and BNIP3-ΔBH3, we demonstrated that the interaction of three proteins Mieap, NIX, and BNIP3 at the MOM is indispensable for opening the pore. We further characterized that the pore induced by these three proteins is not MPTP. Since the pore size of MPTP is too small to explain the mechanism for MALM, we assume that our results are quite reasonable. We hypothesize that the pore induced by the interaction between Mieap, BNIP3, and NIX at the MOM may mediates the translocation of lysosomal proteins from cytoplasm to the inside of mitochondria (Figure 7C). Further careful and continued examination of this hypothesis is needed.

The role of the BH3 domains of BNIP3 and NIX has long remained unclear. In this study, however, we have clearly demonstrated that the BH3 domains of BNIP3 and NIX are indispensable for their interaction with the coiled-coil domains of Mieap. This result implies that the BH3 domains of BNIP3 and NIX play a critical role in MALM. Interestingly, Mieap was localized to the mitochondrial outer membrane and within mitochondria, whereas BNIP3 and NIX were detected only at the mitochondrial outer membrane using IF and proteinase K protection assays. These data suggest that BNIP3 and NIX interact with Mieap via their BH3 domain at the mitochondrial outer membrane and mediate the translocation of Mieap and lysosomal proteins from the cytoplasm to the mitochondrial matrix. Therefore, we propose that the BH3 domain-mediated functions of BNIP3 and NIX are involved in mitochondrial quality control.

During this study, we observed that the *β* form of Mieap was predominantly localized to the mitochondria. Therefore, we speculated that MALM may be regulated by the *β* form of Mieap, and our results support this hypothesis. First, endogenous BNIP3 and NIX proteins were preferentially co-precipitated with the *β* form of the endogenous Mieap protein in immunoprecipitation experiments using cell lysates from the fractionated mitochondria (Figures 1A–1D). Second, the exogenous Mieap *β* form was shown to interact with the exogenous BNIP3 and NIX proteins more strongly than the exogenous Mieap *α* form (Figures 1E, 1F, 2C, 3B). Third, endogenous Mieap *β* was preferentially localized to the mitochondria and detected within the mitochondria in the proteinase K protection assay (Figure 4B). Fourth, Mieap *β* induced the down-regulation of MMP much more effectively than the Mieap *α* form when these proteins were co-expressed with BNIP3 and NIX (Figure 5). Taken together, these data suggest that the *β* form of Mieap might play a specific role in the induction of MALM.

We have previously reported that the p53-Mieap pathway controls mitochondrial quality by repairing or eliminating unhealthy mitochondria via MALM and MIV, respectively [1], [2]. However, how Mieap regulates MALM and MIV remains unclear. The findings of this study suggest that there may be a selective mechanism leading to MALM-mediated repair through Mieap *β* or MIV formation for the elimination of unhealthy mitochondria mediated by Mieap *α*. If the *β* form of Mieap specifically regulates MALM, does the *α* form of Mieap have a specific role in MIV generation? Further investigation is needed to clarify the specific roles of Mieap *α* and *β* in MIV and MALM, respectively.

## Materials and Methods

### Cell lines

The following human cancer cell lines were purchased from the American Type Culture Collection: LS174T and HCT116 (colorectal adenocarcinomas) and A549 (lung cancer). Cells were cultured under the conditions recommended by their depositors.

### Antibodies

The anti-Mieap antibody was prepared as described previously [1]. The other primary antibodies used in this study were mouse monoclonal anti-beta-actin antibody (clone AC-74, Sigma), mouse monoclonal anti-NIX antibody (Abnova), mouse monoclonal anti-BNIP3 antibody (Abcam), mouse monoclonal anti-FLAG antibody (Sigma), goat polyclonal anti-cathepsin B antibody (R & D systems), mouse monoclonal anti-cathepsin D antibody (Novus Biologicals), mouse monoclonal anti-VDAC/Porin antibody (Abcam), rabbit polyclonal anti-TOMM70A antibody (Novus Biologicals), mouse monoclonal anti-ATP synthase alpha antibody (Invitrogen), mouse monoclonal anti-mitofilin antibody (Calbiochem), and mouse monoclonal anti-LAMP1 antibody (BD Pharmingen).

### Immunoprecipitation

To examine the interaction of endogenous Mieap and endogenous NIX and BNIP3, A549 cells were irradiated using γ-rays at 10 Gy, and 48 h after IR, the mitochondria were fractionated as described in the proteinase K protection assay methods. The mitochondrial pellets were lysed on ice for 15 min in 500 µl of NP40 lysis buffer (1% NP40, 150 mM NaCl, 25 mM Tris-HCl, pH 7.6, and complete protease inhibitor cocktail (Roche)).

To examine the interaction of exogenous Mieap and exogenous BNIP3 and NIX, the various forms of Mieap, BNIP3 and NIX were expressed in HCT116 cells through infection with adenoviral vectors expressing Mieap and Mieap-mutants at MOIs of 5 or transfection with plasmids expressing the N-FLAG-tagged NIX, NIX-mutants, BNIP3, and BNIP3-mutants. At 36 h after infection, the cells were lysed on ice for 15 min in 500 µl of NP40 lysis buffer.

After lysis, cellular debris was removed using centrifugation at 12,000×g for 15 min, and the supernatant was collected. The supernatant was precleared by absorbing it with normal IgG and 20 µl of protein-A or Protein-G sepharose beads for 1 h at 4°C. The beads were removed using centrifugation, and the supernatant was subjected to IP by adding 1 µg rabbit polyclonal anti-Mieap antibody, 1 µg mouse monoclonal anti-NIX antibody, 1 µg mouse monoclonal anti-BNIP3 antibody or 1 µg mouse monoclonal anti-FLAG antibody. Normal rabbit IgG (Santa Cruz Biotechnology) and normal mouse IgG (Santa Cruz Biotechnology) were used as negative IP controls. The antibody mixtures were allowed to react on a rotating device overnight at 4°C. Protein-A or Protein-G sepharose beads were added, and the mixtures were incubated for an additional 2 h at 4°C. The beads were washed five times in cold lysis buffer, and the immune complexes were released from the beads by boiling in 2× Laemmli sample buffer. Samples were loaded onto 10–15% SDS-PAGE gels, and the electrophoresed proteins were subjected to western blot analysis using anti-Mieap, anti-NIX, anti-BNIP3, or anti-FLAG antibodies.

### Immunocytochemistry

For immunocytochemistry, cells were grown on eight-well chamber slides (2×10^4^ cells/well) at 37°C in conventional culture medium and fixed in 2% paraformaldehyde for 10 min at room temperature. Slides were incubated with 0.1% Triton X-100 in phosphate-buffered saline (PBS) for 3 min and washed three times with PBS at room temperature. Cells were blocked with 3% bovine serum albumin (BSA) in PBS for 1 h and incubated with primary antibodies for 1 h at room temperature. After being washed three times with PBS, slides were incubated with fluorescein isothiocyanate (FITC)-conjugated or Alexa Fluor 546-conjugated secondary antibodies for 1 h at room temperature. Slides were treated with 1 µM TO-PRO-3 (Invitrogen) for 15 min to stain the nuclei and washed four times with PBS. Slides were mounted with VECTASHIELD H-1000 (Vector Laboratories) and observed under an Olympus IX70 (Olympus) inverted fluorescence microscope coupled to a Radiance 2000 laser-scanning confocal system (Bio-Rad).

### Establishment of BNIP3-KD and NIX/BNIP3 double-KD cell lines using RNA interference

We established BNIP3-KD and NIX/BNIP3 double-KD cell lines in A549 cells, as described previously [1], [2]. BNIP3 expression was inhibited in the A549 cell line through retroviral expression under the H1 promoter of short-hairpin RNA (shRNA) (BNIP3-KD: 5′-gatccccACACGAGCGTCATGAAGAAttcaagagaTTCTTCATGACGCTCGTGTtttttggaaa-3′) against the BNIP3 sequence. To generate the NIX/BNIP3 double-KD cell line, the NIX-KD A549 cells were infected with another retroviral vector containing the U6 promoter (Takara) and expressing the BNIP3-KD shRNA. We also established control cell lines through infection with an empty retroviral vector (Control).

### DNA-damaging treatments

Cells were seeded 12 h before treatment and were 60–70% confluent at the time of the treatment. To induce MALM, the cells were γ-irradiated at 10 Gy or 60 Gy using a ^60^Co source.

### Subcellular fractionation and proteinase K protection assay

The experiment was performed as described previously [1]. HCT116 cells were co-infected with Ad-Mieap *β*, Ad-FALG-NIX, and Ad-FLAG-BNIP3 at an MOI of 5, and 2 h after the infection, the cells were γ-irradiated at 10 Gy and collected 30 h after IR. A549 and LS174T cells were γ-irradiated at 10 Gy and collected at 48 h after IR. The cells were suspended in cold homogenization buffer (20 mM HEPES, pH 7.4, and 250 mM sucrose) at 2×10^7^ cells/ml and homogenized using Dounce homogenization on ice. The homogenized samples were centrifuged at 800×g at 4°C for 10 min to pellet the nuclear fraction. The centrifugation was repeated, and the resulting supernatant was centrifuged at 10,000×g at 4°C for 10 min to obtain a mitochondrial pellet and cytosolic supernatant. The mitochondrial pellets were centrifuged again with fresh homogenization buffer, and the pellets were suspended in 25 mM Tris-HCl, pH 7.4, and 150 mM NaCl to react with the protease. The mitochondrial suspensions were treated or not with 20 µg/ml proteinase K for 20 min on ice. Then, proteinase K was inactivated using 2 mM PMSF for 10 min on ice. The samples were dissolved in Laemmli buffer for SDS–PAGE, and western blotting was performed using primary antibodies against the indicated proteins.

### Quantitative analysis of the accumulation of Mieap and lysosomes within mitochondria

The control, BNIP3-KD, NIX-KD, and NIX/BNIP3 double-KD A549 cells were seeded in eight-well chamber slides (2×10^4^ cells/well) at 37°C in conventional medium. The cells were infected with Ad-DsRed-Mito at an MOI of 30 and γ-irradiated at 60 Gy 12 h after infection. On day 3 after IR, the cells were subjected to IF analysis. The overlap between Mieap and mitochondrial signals (yellow area) or between the mitochondrial and lysosomal signals (yellow area) was analyzed using LuminaVision image analysis software in 300–400 cells as previously described [2]. The total areas of yellow intensity in 300–400 cells were determined using optimal threshold parameters and calculated using LuminaVision image analysis software. The intensity is presented as the average of the calculated values per cell with error bars.

### Mitochondrial membrane potential

To examine MMP, the cells were incubated with 10 µM rhodamine 123 fluorescent dye (Sigma) in PBS for 15 min at 37°C in the dark. Next, the cells were collected using centrifugation, washed in PBS, and resuspended in PBS. The stained cells were immediately analyzed using a FACS Calibur Flow Cytometer (Becton Dickinson).

To evaluate the involvement of MPTP, cyclosporine A (WAKO) was used to inhibit MPTP.

### Cell death assay

Live and dead cells were counted using the trypan blue dye exclusion method.

Apoptotic cells were evaluated by measuring caspase-3 activity. Caspase-3 activity was examined using tetrapeptide p-nitroanilide (pNA) substrates. 10 µl of the cell lysates in 96-well plate was incubated with 90 µl of reaction buffer (50 mM HEPES, ph 7.4, 100 mM NaCl, 0.1% CHAPS, 1 mM EDTA, and 10% glycerol) containing 250 µM of pNA substrate (Ac-DEVD-pNA: Sigma). Absorbance at 405 nm was measured immediately and 2 h after using a microtiter plate reader (Molecular Devices Inc.).

To examine the release of cytochrome c from mitochondria, IF experiment with mouse monoclonal anti-cytochrome c antibody (clone 6H2.B4, Cell Sciences) was carried out.

## Supporting Information

Figure S1 **The NIX and BNIP3 expressions are inducible by ionizing radiation.** The cont, NIX-KD, and BNIP3-KD cells of A549 were treated or not treated by ionizing radiation (IR) at 10 Gy. At 48 h after IR, the cell lysates were subjected to western blot analysis. The endogenous NIX and BNIP3 proteins were detected with anti-NIX and anti-BNIP3 antibodies.
(TIF)

Figure S2 **The subcellular localization of the NIX and BNIP3 mutants.** The IF experiment was performed using the anti-FLAG antibody (NIX or BNIP3: green) and DsRed-mito (Mito: red). HCT116 cells were transfected with plasmids expressing the indicated NIX and BNIP3 mutants. Scale bar = 10 µm.
(TIF)

Figure S3 **The NIX and BNIP3 expressions are inhibited in the BNIP3-KD, NIX-KD, and NIX/BNIP3-KD cells.** The indicated cell lines were treated by IR at 60 Gy. At 48 h after IR, the cell lysates were subjected to western blot analysis. The endogenous NIX and BNIP3 proteins were detected with anti-NIX and anti-BNIP3 antibodies.
(TIF)

Figure S4 **The exogenous NIX and BNIP3 were sufficiently expressed in the HCT116 cells by the infection with Ad-NIX or Ad-BNIP3 at MOIs of 5, 30, and 100.** The HCT116 cells were infected with Ad-NIX or Ad-BNIP3 at MOIs of 5, 30, and 100. At 48 h and 96 h after the infection, the cell lysates were subjected to western blot analysis. The exogenous NIX and BNIP3 proteins were detected with anti-NIX and anti-BNIP3 antibodies.
(TIF)

Figure S5 **Overexpressions of BNIP3 and NIX do not induce the MMP reduction and cell death.** HCT116 cells were infected with Ad-NIX, Ad-BNIP3 and Ad-LacZ at MOIs of 5, 30, 100, or Ad-p53 at a MOI of 5. At 48 h and 96 h after the infection, the MMP reduction was determined through the percentage of rhodamine-negative cells (A), and cell death was evaluated by carrying out trypan blue exclusion assay (B), and by measuring the caspase-3 activity (C). Ad-p53 at a MOI of 5 was used as a positive control for cell death. The average values of three independent experiments are presented; the error bars indicate 1 SD. p<0.01 (*) was considered statistically significant between the Ad-LacZ infection at a MOI of 5 and the Ad-p53 infection at a MOI of 5 (A, B, and C).
(TIF)

Figure S6 **Overexpressions of BNIP3 and NIX do not induce cell death.** The morphology in the experiment of Figure S5 was shown. Scale bar = 200 µm.
(TIF)

Figure S7 **The MMP reduction induced by the triple co-expression of Mieap, BNIP3, and NIX is not related to cell death.** The morphology in the experiment of Figure 6 was shown. Scale bar = 200 µm.
(TIF)

Figure S8 **The MMP reduction induced by the triple co-expression of Mieap, BNIP3, and NIX does not induce the release of cytochrome c from the mitochondria.** The subcellular localization of cytochrome c was shown in the experiment of Figure 6. Scale bar = 20 µm.
(TIF)

## References

[1] Miyamoto Y, Kitamura N, Nakamura Y, Futamura M, Miyamoto T (2011). Possible existence of lysosome-like organella within mitochondria and its role in mitochondrial quality control.. PLoS ONE.

[2] Kitamura N, Nakamura Y, Miyamoto Y, Miyamoto T, Kabu K (2011). Mieap, a p53-inducible protein, controls mitochondrial quality by repairing or eliminating unhealthy mitochondria.. PLoS ONE.

[3] Zhang J, Ney PA (2009). Role of BNIP3 and NIX in cell death, autophagy, and mitophagy.. Cell Death Differ.

[4] Boyd JM, Malstrom S, Subramanian T, Venkatesh LK, Schaeper U (1994). Adenovirus E1B 19 kDa and Bcl-2 proteins interact with a common set of cellular proteins.. Cell.

[5] Vande Velde C, Cizeau J, Dubik D, Alimonti J, Brown T (2000). BNIP3 and genetic control of necrosis-like cell death through the mitochondrial permeability transition pore.. Mol Cell Biol.

[6] Cizeau J, Ray R, Chen G, Gietz RD, Greenberg AH (2000). The C. elegans orthologue ceBNIP3 interacts with CED-9 and CED-3 but kills through a BH3- and caspase-independent mechanism.. Oncogene.

[7] Ray R, Chen G, Vande Velde C, Cizeau J, Park JH (2000). BNIP3 heterodimerizes with Bcl-2/Bcl-X(L) and induces cell death independent of a Bcl-2 homology 3 (BH3) domain at both mitochondrial and nonmitochondrial sites.. J Biol Chem.

[8] Dorn GW (2010). Mitochondrial pruning by Nix and BNip3: an essential function for cardiac-expressed death factors.. J Cardiovasc Transl Res.

[9] Chen G, Ray R, Dubik D, Shi L, Cizeau J (1997). The E1B 19K/Bcl-2-binding protein Nip3 is a dimeric mitochondrial protein that activates apoptosis.. J Exp Med.

[10] Kubasiak LA, Hernandez OM, Bishopric NH, Webster KA (2002). Hypoxia and acidosis activate cardiac myocyte death through the Bcl-2 family protein BNIP3.. Proc Natl Acad Sci USA.

[11] Kim JY, Cho JJ, Ha J, Park JH (2002). The carboxy terminal C-tail of BNip3 is crucial in induction of mitochondrial permeability transition in isolated mitochondria.. Arch Biochem Biophys.

[12] Bocharov EV, Pustovalova YE, Pavlov KV, Volynsky PE, Goncharuk MV (2007). Unique dimeric structure of BNip3 transmembrane domain suggests membrane permeabilization as a cell death trigger.. J Biol Chem.

[13] Chen G, Cizeau J, Vande Velde C, Park JH, Bozek G (1999). Nix and Nip3 form a subfamily of pro-apoptotic mitochondrial proteins.. J Biol Chem.

[14] Kim H, Rafiuddin-Shah M, Tu HC, Jeffers JR, Zambetti GP (2006). Hierarchical regulation of mitochondrion-dependent apoptosis by BCL-2 subfamilies.. Nat Cell Biol.

