# Supplementary figures and images for: BNIP3 and NIX Mediate Mieap-Induced Accumulation of Lysosomal Proteins within Mitochondria
Source: PLoS One. 2012 Jan 26;7(1):e30767. doi: 10.1371/journal.pone.0030767 (PMC3266916; doi:10.1371/journal.pone.0030767)

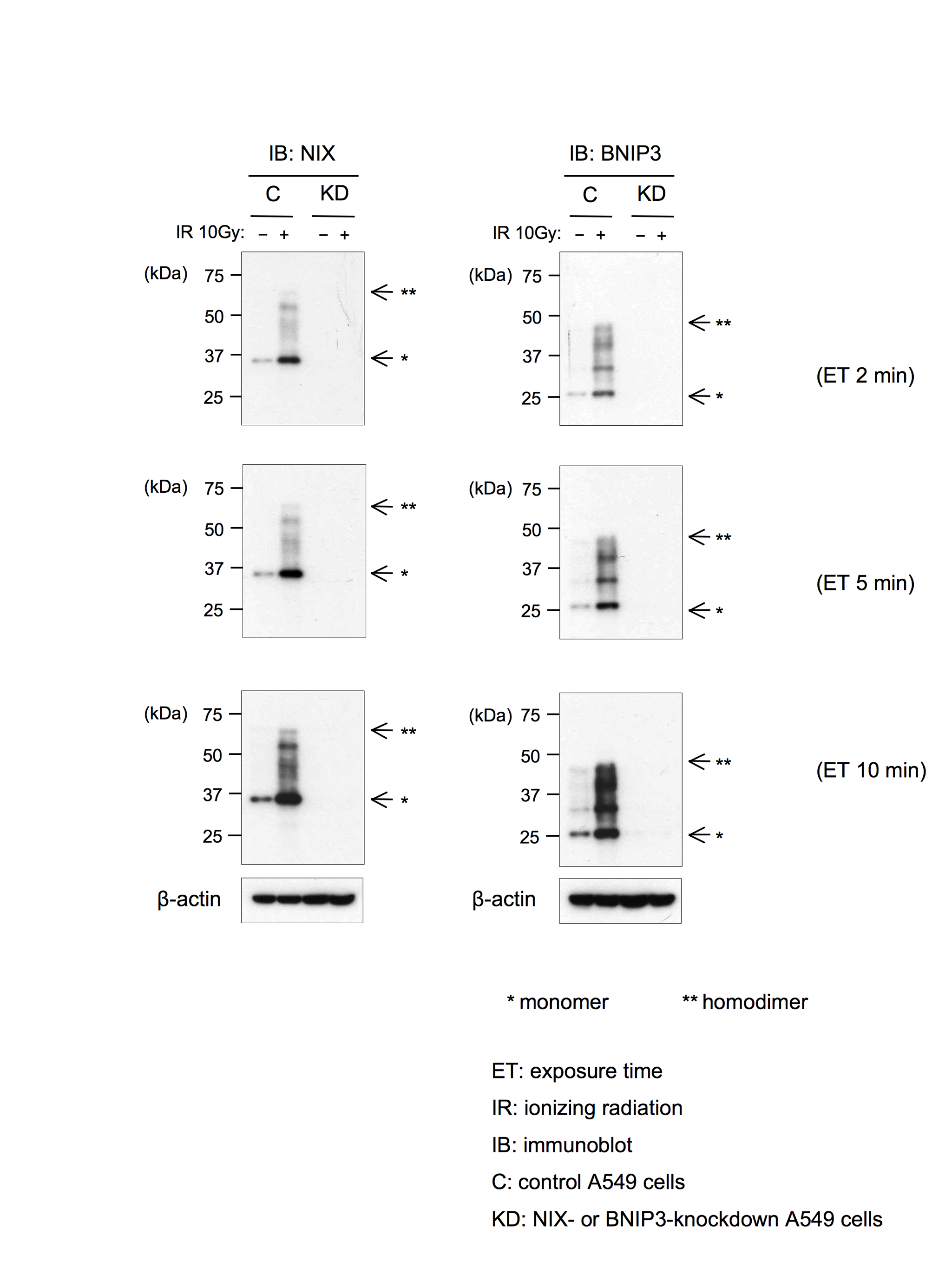

Supplement: Figure S1 — The NIX and BNIP3 expressions are inducible by ionizing radiation. The cont, NIX-KD, and BNIP3-KD cells of A549 were treated or not treated by ionizing radiation (IR) at 10 Gy. At 48 h after IR, the cell lysates were subjected to western blot analysis. The endogenous NIX and BNIP3 proteins were detected with anti-NIX and anti-BNIP3 antibodies. (TIF) [file pone.0030767.s001.tif]

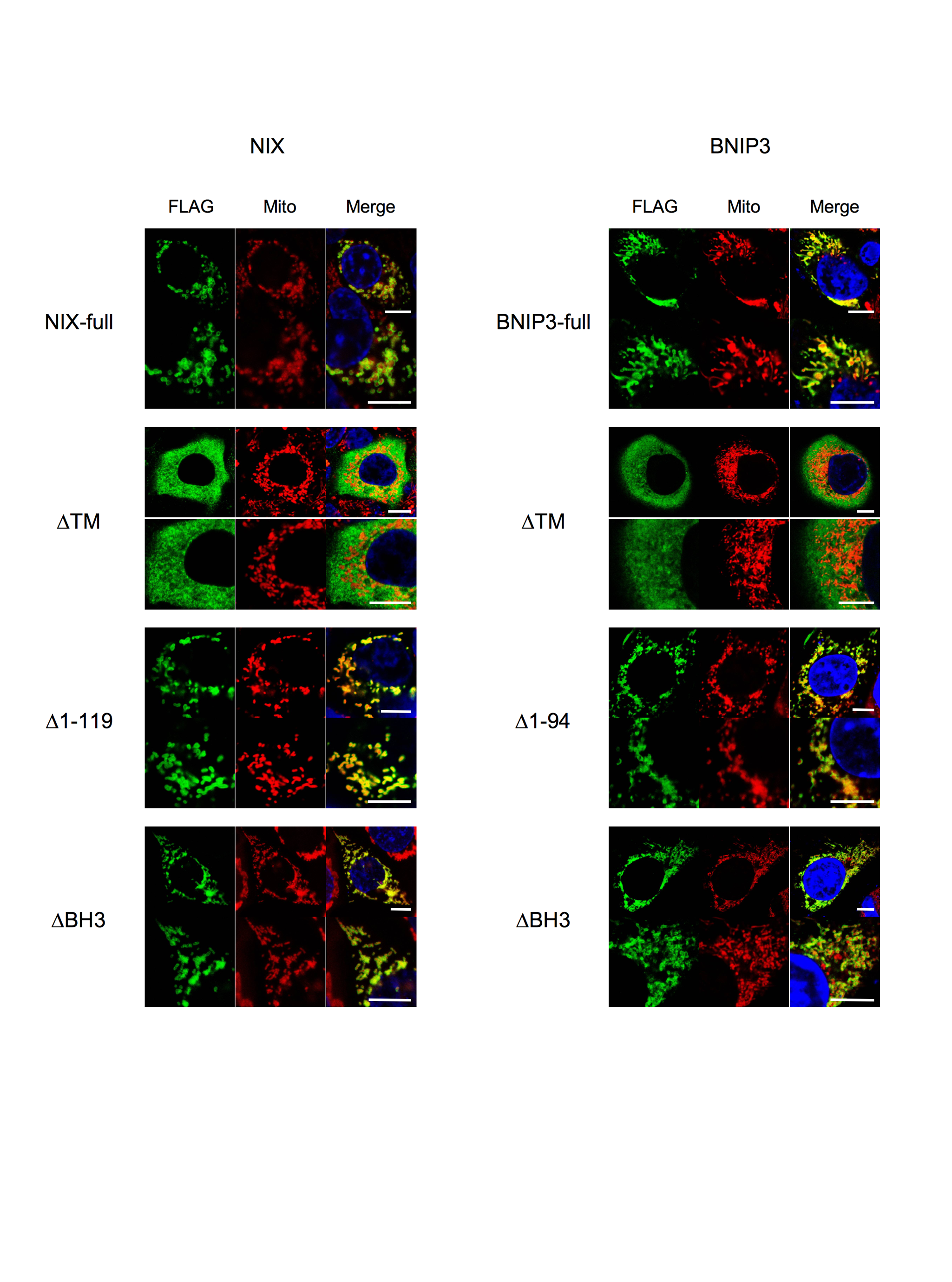

Supplement: Figure S2 — The subcellular localization of the NIX and BNIP3 mutants. The IF experiment was performed using the anti-FLAG antibody (NIX or BNIP3: green) and DsRed-mito (Mito: red). HCT116 cells were transfected with plasmids expressing the indicated NIX and BNIP3 mutants. Scale bar = 10 µm. (TIF) [file pone.0030767.s002.tif]

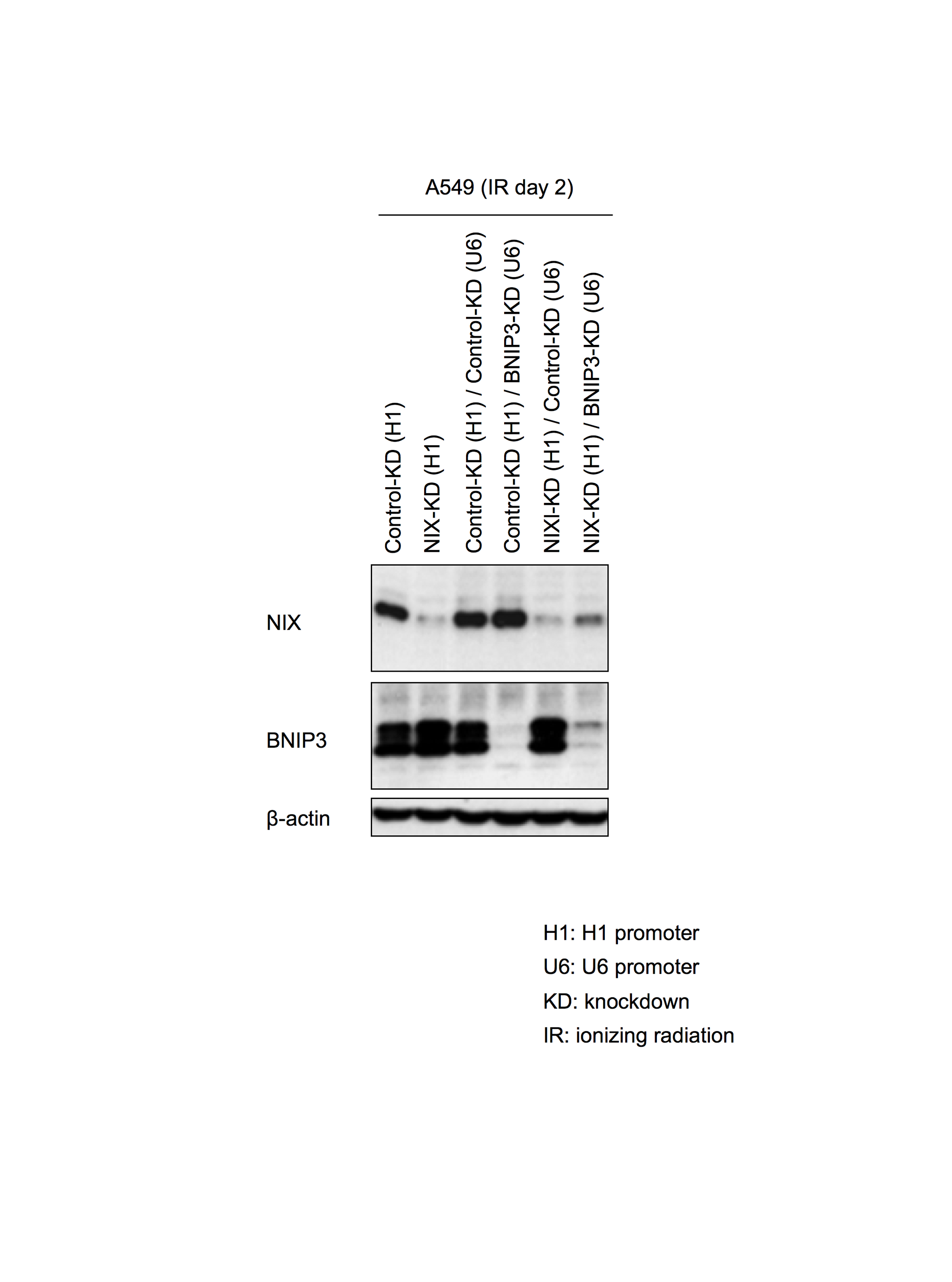

Supplement: Figure S3 — The NIX and BNIP3 expressions are inhibited in the BNIP3-KD, NIX-KD, and NIX/BNIP3-KD cells. The indicated cell lines were treated by IR at 60 Gy. At 48 h after IR, the cell lysates were subjected to western blot analysis. The endogenous NIX and BNIP3 proteins were detected with anti-NIX and anti-BNIP3 antibodies. (TIF) [file pone.0030767.s003.tif]

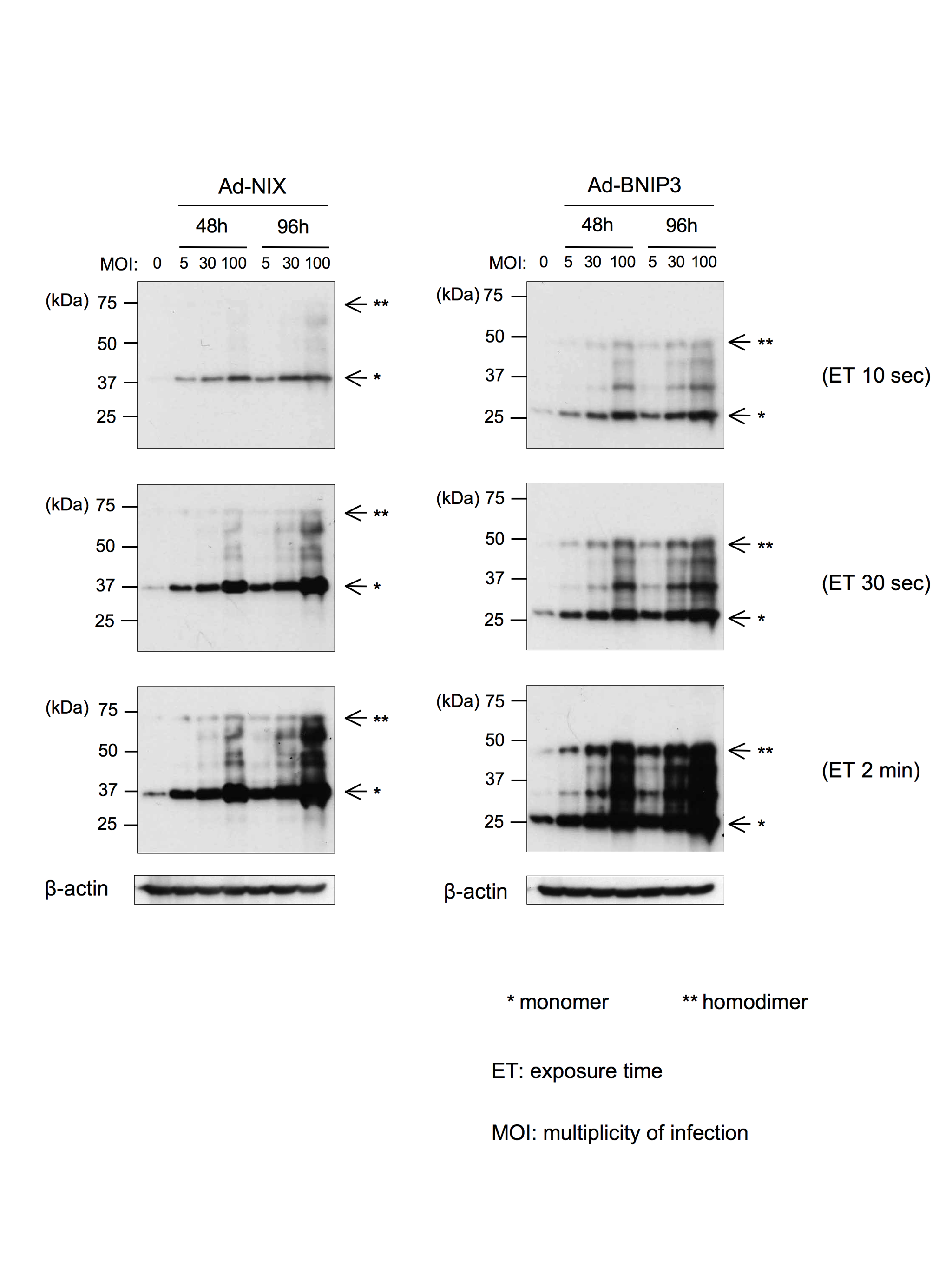

Supplement: Figure S4 — The exogenous NIX and BNIP3 were sufficiently expressed in the HCT116 cells by the infection with Ad-NIX or Ad-BNIP3 at MOIs of 5, 30, and 100. The HCT116 cells were infected with Ad-NIX or Ad-BNIP3 at MOIs of 5, 30, and 100. At 48 h and 96 h after the infection, the cell lysates were subjected to western blot analysis. The exogenous NIX and BNIP3 proteins were detected with anti-NIX and anti-BNIP3 antibodies. (TIF) [file pone.0030767.s004.tif]

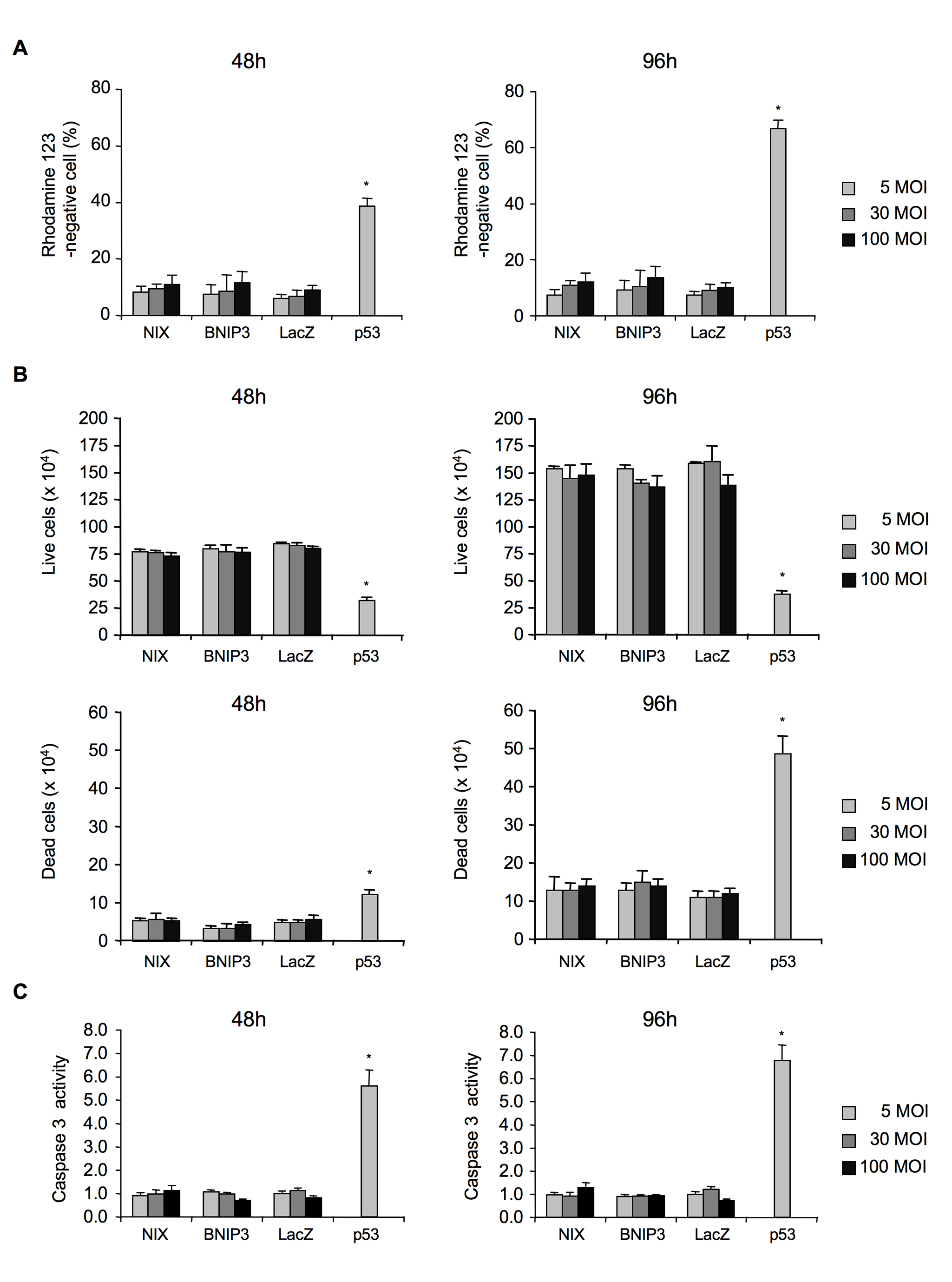

Supplement: Figure S5 — Overexpressions of BNIP3 and NIX do not induce the MMP reduction and cell death. HCT116 cells were infected with Ad-NIX, Ad-BNIP3 and Ad-LacZ at MOIs of 5, 30, 100, or Ad-p53 at a MOI of 5. At 48 h and 96 h after the infection, the MMP reduction was determined through the percentage of rhodamine-negative cells (A), and cell death was evaluated by carrying out trypan blue exclusion assay (B), and by measuring the caspase-3 activity (C). Ad-p53 at a MOI of 5 was used as a positive control for cell death. The average values of three independent experiments are presented; the error bars indicate 1 SD. p<0.01 (*) was considered statistically significant between the Ad-LacZ infection at a MOI of 5 and the Ad-p53 infection at a MOI of 5 (A, B, and C). (TIF) [file pone.0030767.s005.tif]

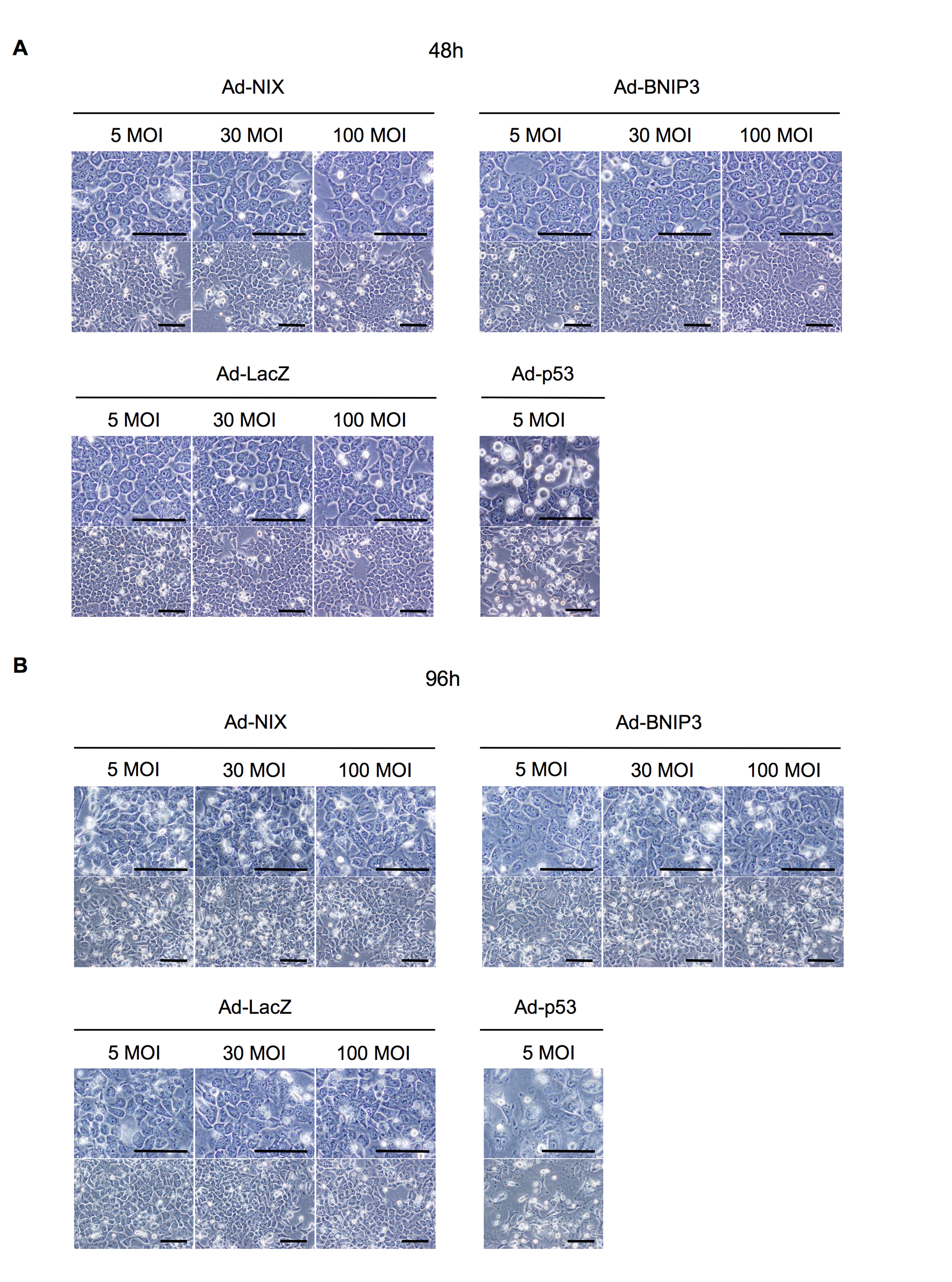

Supplement: Figure S6 — Overexpressions of BNIP3 and NIX do not induce cell death. The morphology in the experiment of Figure S5 was shown. Scale bar = 200 µm. (TIF) [file pone.0030767.s006.tif]

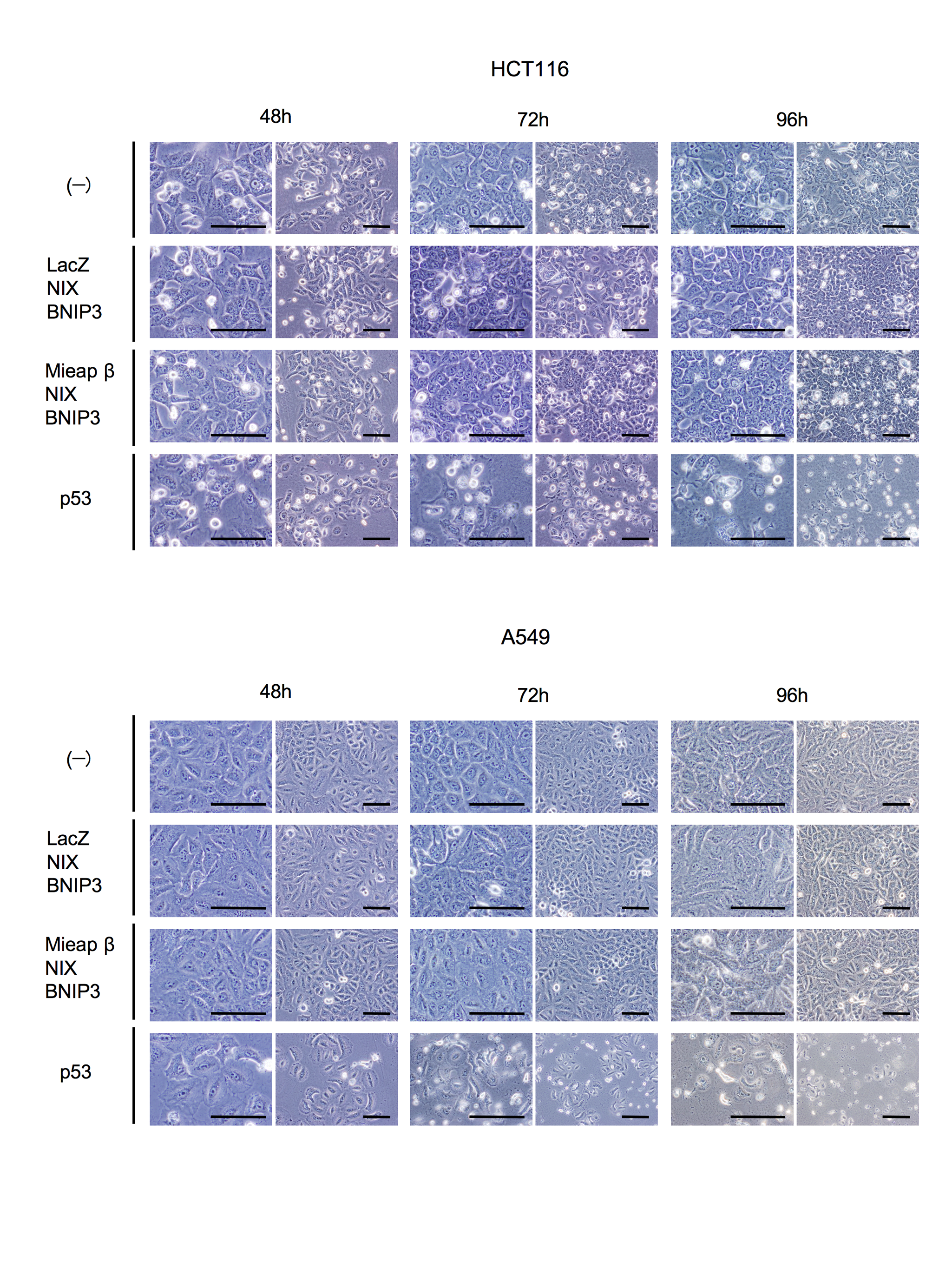

Supplement: Figure S7 — The MMP reduction induced by the triple co-expression of Mieap, BNIP3, and NIX is not related to cell death. The morphology in the experiment of Figure 6 was shown. Scale bar = 200 µm. (TIF) [file pone.0030767.s007.tif]

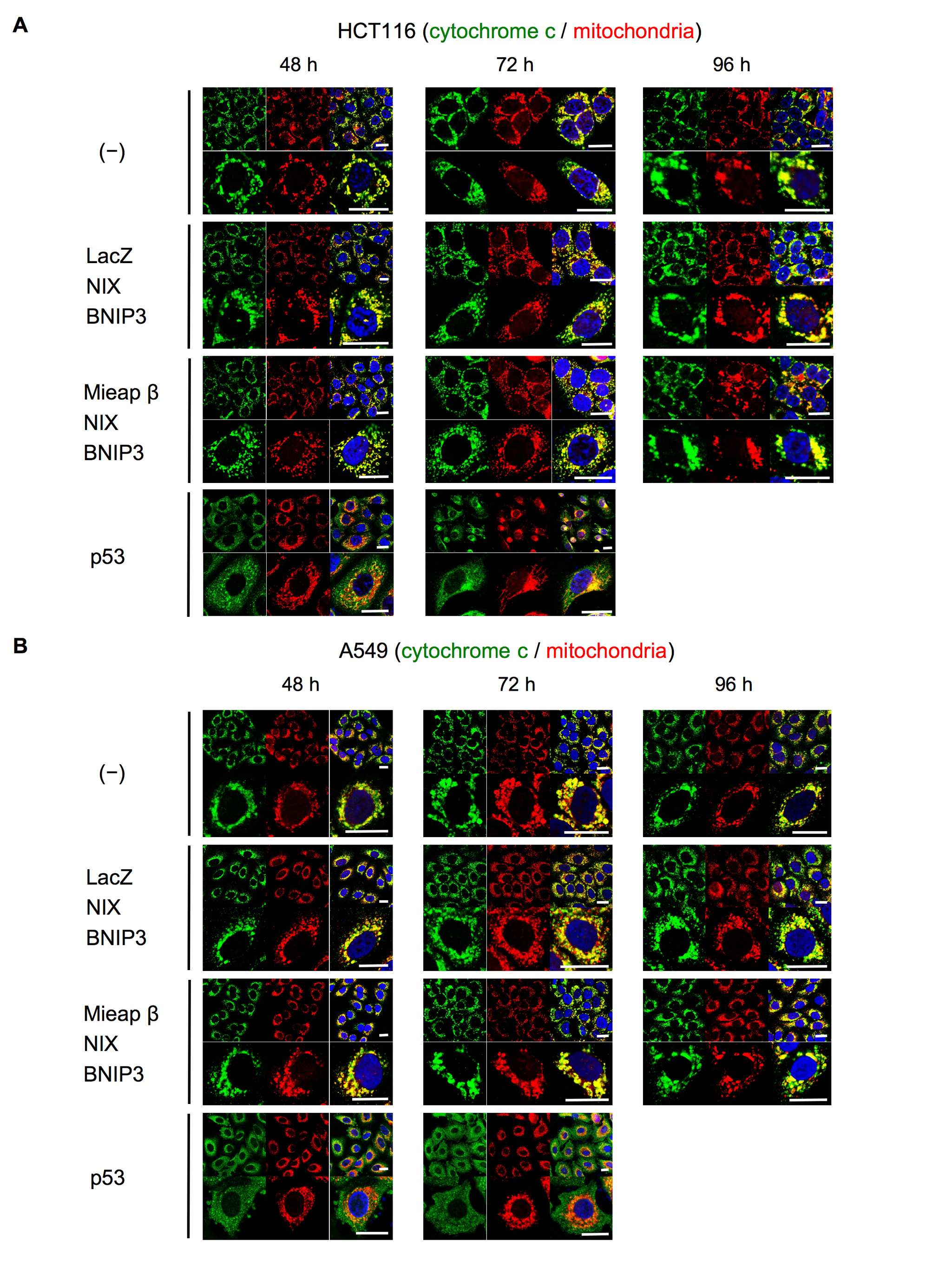

Supplement: Figure S8 — The MMP reduction induced by the triple co-expression of Mieap, BNIP3, and NIX does not induce the release of cytochrome c from the mitochondria. The subcellular localization of cytochrome c was shown in the experiment of Figure 6. Scale bar = 20 µm. (TIF) [file pone.0030767.s008.tif]
